# Structural insights into mechanisms of Argonaute protein-associated NADase activation in bacterial immunity

**DOI:** 10.1038/s41422-023-00839-7

**Published:** 2023-06-13

**Authors:** Xiaoshen Wang, Xuzichao Li, Guimei Yu, Lingling Zhang, Chendi Zhang, Yong Wang, Fumeng Liao, Yanan Wen, Hang Yin, Xiang Liu, Yong Wei, Zhuang Li, Zengqin Deng, Heng Zhang

**Affiliations:** 1 National key laboratory of blood science, The Province and Ministry Co-sponsored Collaborative Innovation Center for Medical Epigenetics, Key Laboratory of Immune Microenvironment and Disease (Ministry of Education), Haihe Laboratory of Cell Ecosystem, Tianjin Institute of Immunology, Department of Biochemistry and Molecular Biology, School of Basic Medical Sciences, Tianjin Medical University, Tianjin, China; 2 State Key Laboratory of Biocatalysis and Enzyme Engineering, School of Life Sciences, Hubei University, Wuhan, Hubei China; 3 Center for Antiviral Research, Wuhan Institute of Virology, Chinese Academy of Sciences, Wuhan, Hubei China; 4 University of Chinese Academy of Sciences, Beijing, China; 5 Department of Pharmacology, School of Basic Medical Sciences, Tianjin Medical University, Tianjin, China; 6 State Key Laboratory of Medicinal Chemical Biology, Frontiers Science Center for Cell Responses, College of Life Sciences, Nankai University, Tianjin, China; 7 The Cancer Hospital of the University of Chinese Academy of Sciences (Zhejiang Cancer Hospital), Institute of Basic Medicine and Cancer (IBMC), Chinese Academy of Sciences, Hangzhou, Zhejiang China; 8 Hubei Jiangxia Laboratory, Wuhan, Hubei China

**Keywords:** Electron microscopy, Molecular biology

## Abstract

Nicotinamide adenine dinucleotide (NAD^+^) is a central metabolite in cellular processes. Depletion of NAD^+^ has been demonstrated to be a prevalent theme in both prokaryotic and eukaryotic immune responses. Short prokaryotic Argonaute proteins (Agos) are associated with NADase domain-containing proteins (TIR-APAZ or SIR2-APAZ) encoded in the same operon. They confer immunity against mobile genetic elements, such as bacteriophages and plasmids, by inducing NAD^+^ depletion upon recognition of target nucleic acids. However, the molecular mechanisms underlying the activation of such prokaryotic NADase/Ago immune systems remain unknown. Here, we report multiple cryo-EM structures of NADase/Ago complexes from two distinct systems (TIR-APAZ/Ago and SIR2-APAZ/Ago). Target DNA binding triggers tetramerization of the TIR-APAZ/Ago complex by a cooperative self-assembly mechanism, while the heterodimeric SIR2-APAZ/Ago complex does not assemble into higher-order oligomers upon target DNA binding. However, the NADase activities of these two systems are unleashed via a similar closed-to-open transition of the catalytic pocket, albeit by different mechanisms. Furthermore, a functionally conserved sensor loop is employed to inspect the guide RNA–target DNA base pairing and facilitate the conformational rearrangement of Ago proteins required for the activation of these two systems. Overall, our study reveals the mechanistic diversity and similarity of Ago protein-associated NADase systems in prokaryotic immune response.

## Introduction

Argonaute proteins (Agos), the nucleic acid-guided nucleases, are key players in the DNA/RNA-interference pathways in all three domains of life.^1^ The eukaryotic Ago (eAgo) proteins can be classified into two clades, AGO-clade and PIWI-clade, both of which are involved in the guide RNA (gRNA)-mediated RNA targeting.^2^ The ubiquitously expressed AGO-clade proteins primarily associate with small interfering RNAs (siRNAs) or microRNAs (miRNAs) to regulate the expression of target genes.^3^ The mostly germline-specific PIWI-clade proteins interact with PIWI-interacting RNAs (piRNAs) and play essential roles in silencing transposable genetic elements.^4^ A highly conserved lysine is used to recognize the 5′-phosphate moiety of gRNA in the AGO-clade proteins, while PIWI-clade Ago proteins utilize a metal ion to coordinate the 5′-phosphate group of gRNA.^5^ The typically pre-arranged seed region, a stretch of nucleotides spanning positions 2–8 in the gRNA, plays a critical role in target binding and cleavage for eAgos.^6^

Similar to eAgo proteins, long prokaryotic Ago (pAgo) proteins adopt a bilobed four-domain organization, including the N, PAZ, MID, and PIWI domains.^6,7^ Recognition of the 5′-phosphate group of gRNA in the long pAgo systems relies on a metal ion, reminiscent of that in the PIWI-clade proteins.^8^ In contrast to eAgos, mismatches in the seed region have moderate effects in long pAgos.^9^ Besides long pAgo proteins, another clade of pAgo proteins consists of only MID and PIWI domains, and is therefore termed as short pAgos.^10–12^ Notably, nuclease activity is absent due to the lack of the DEDX catalytic tetrad in the PIWI domain of short pAgos. Instead, short pAgos are typically associated with APAZ (analog of PAZ) domain-containing proteins encoded in the same operon. Interestingly, the APAZ domain, which is homologous to the N domain (rather than the PAZ domain) of long pAgo proteins, is usually fused to enzymatic domains such as TIR, SIR2, MRR and DUF4365.^7,10^

The TIR and SIR2 domains bearing NADase activity have been widely implicated in the cell death signaling pathway through NAD^+^ degradation in both prokaryotic and eukaryotic cells.^13–15^ After axon injury, the mammalian TIR domain-containing protein SARM1 cleaves NAD^+^ to cause axon degeneration.^16,17^ In plants and bacteria, the NADase activity of TIR domain provides immunity against pathogen infection.^18–27^ The self-association of the TIR domain is required to elicit the NADase activity by a conserved BB-loop transition mechanism.^19,21,23,28^ Another NAD-consuming module, SIR2, is also found to be involved in bacterial immune systems, such as the Thoeris antiphage defense system.^27^ In these cell death pathways, the TIR or SIR2 domain could convert NAD^+^ into Nam, ADP-ribose (ADPR), cyclic ADPR (cADPR), or variant cADPR (v-cADPR).^27,29^

Most recently, short pAgos were shown to interact with NADase-APAZ fusion proteins (TIR-APAZ and SIR2-APAZ) to protect bacteria against invading DNA, such as bacteriophages and plasmids.^30–32^ Cell death is induced by NADase activation upon the recognition of target single-stranded DNA (ssDNA) to inhibit phage/plasmid infection. However, the mechanism underlying the nucleic acid-guided NADase activation is not clear. Here, we report multiple cryo-EM structures of TIR-APAZ/Ago and SIR2-APAZ/Ago complexes at different functional states. Combining biochemical and mutagenesis studies, we reveal both diverse and conserved features of the pAgo-associated NADase systems in bacterial immune response. Our work provides the first structural understanding of the short pAgo-associated NADase, and also builds the foundation for further mechanistic characterization of short pAgo and NAD^+^ depletion in bacterial immunity.

## Results

### Cryo-EM structure of TIR-APAZ/Ago–gRNA complex

**Fig. 1:**
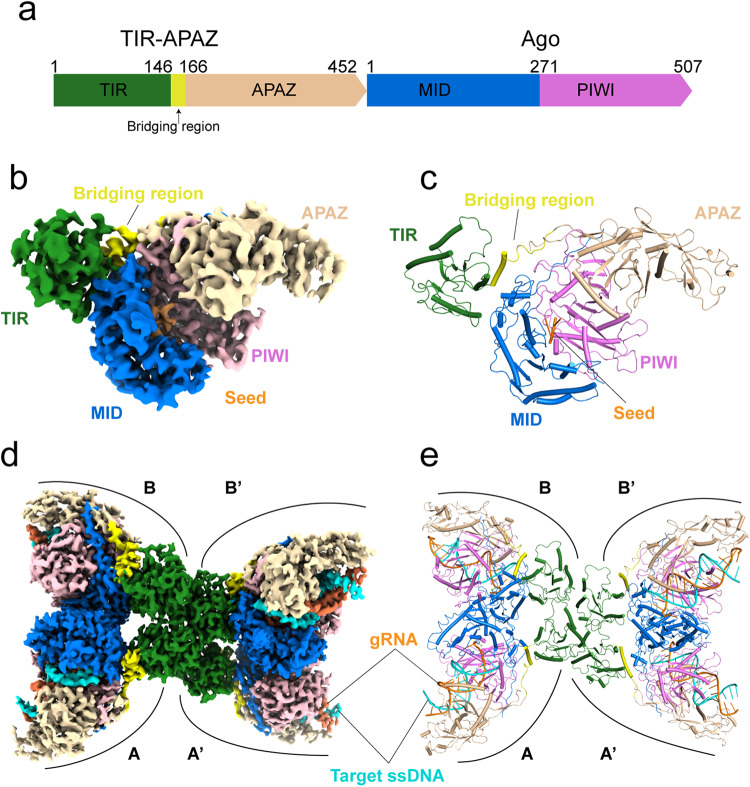
Cryo-EM structures of TIR-APAZ/Ago system in target-free and target-bound states. **a** Domain organization of the TIR-APAZ and Ago proteins. The TIR domain, bridging region, APAZ domain, MID domain and PIWI domain are colored in green, yellow, wheat, blue and pink, respectively. **b** Cryo-EM density map of the gRNA-bound TIR-APAZ/Ago complex. The densities of domains are colored as in **a**. The seed region of gRNA is colored in orange. **c** The atomic model of the TIR-APAZ/Ago–gRNA RNP complex. The same color scheme as in **b** is used. **d** Cryo-EM density map of the target ssDNA-bound TIR-APAZ/Ago–gRNA quaternary complex. The letters A, A’, B and B’ indicate the four units in the tetramer. The target ssDNA is colored in cyan. **e** The atomic model of the target ssDNA-bound TIR-APAZ/Ago–gRNA quaternary complex.

To investigate the interaction mode between TIR-APAZ and Ago proteins, we first co-purified the binary complex from *Maribacter polysiphoniae* (termed TIR-APAZ/Ago complex hereinafter, also known as SPARTA)^30^ and determined its cryo-EM structure in the presence of gRNA (Fig. 1a, b; Supplementary information, Fig. S1 and Table S1). We were able to build the structure of most regions of the TIR-APAZ/Ago complex, with the exception of the C-terminal region (aa 419–452) of TIR-APAZ and residues 161–200 of the Ago protein. Although the majority of the gRNA could not be resolved, two nucleotides (2G–3A), defined as the seed region, could be clearly seen (Fig. 1b, c; Supplementary information, Fig. S2a). This observed seed region is much shorter in length than the seed sequences pre-organized by other previously reported eAgos and pAgos.^6^

In the TIR-APAZ/Ago–gRNA ribonucleoprotein (RNP) complex structure, the Ago protein is bracketed by the separated TIR and APAZ domains of TIR-APAZ protein, which are connected by a helix (aa 146–158) and the following loop, termed here as the bridging region (Fig. 1a–c). The TIR domain exhibits a flavodoxin-like fold, consisting of a central five-stranded parallel β-sheet flanked by four α-helices^15,33^ (Supplementary information, Fig. S2b). The TIR domain mainly contacts the MID region of Ago, while the APAZ domain primarily associates with the PIWI segment of Ago, adopting a bilobed architecture reminiscent of the PIWI-clade Agos^34,35^ (Fig. 1b, c). Ago protein, together with the APAZ domain of APAZ-TIR protein, creates a positively charged channel to potentially accommodate the gRNA–target DNA heteroduplex (Supplementary information, Fig. S2c). The RNP complex structure is nearly identical to the apo TIR-APAZ/Ago structure predicted by AlphaFold^36^ (Supplementary information, Fig. S2d), with an RMSD of 0.936 Å, suggesting that gRNA has little impact on the configuration of TIR-APAZ/Ago complex, possibly due to the short pre-organized seed sequence or the lack of PAZ domain, which is absent in short pAgo system.

### Cryo-EM structure of TIR-APAZ/Ago–gRNA–DNA complex

The TIR domains in both apo TIR-APAZ/Ago and RNP complexes are catalytically inactive, but target ssDNA binding could efficiently activate the NADase activity.^30^ To elucidate the molecular basis for the activation of TIR-APAZ/Ago complex, we reconstituted the target ssDNA-bound quaternary complex and determined its cryo-EM structure at a resolution of 2.95 Å (Fig. 1d; Supplementary information, Fig. S3 and Table S1). The well-defined EM density allowed us to unambiguously build an atomic model (Fig. 1e). The C-terminal segment in APAZ domain and a stretch of amino acids in MID domain could not be traced, similar to our observation in the RNP structure, likely owing to the intrinsic flexibility of these regions. Deletion of these regions (Δ171–200 and Δ418–452) had little effect on the NADase activity (Supplementary information, Fig. S4a), indicating that these two flexible parts contribute little to TIR-APAZ/Ago activation. As expected, the gRNA–DNA hybrid is clamped between Ago protein and the APAZ domain of TIR-APAZ protein (Fig. 1d, e; Supplementary information, Fig. S4b).

The TIR-APAZ/Ago–gRNA–DNA complex displays a “butterfly-shaped” architecture with four copies of the TIR-APAZ/Ago heterodimer (Fig. 1d, e), consistent with the previous results that the engagement of gRNA–DNA duplex could induce higher-order oligomerization of the TIR-APAZ/Ago complex.^30^ The four TIR-APAZ/Ago protomers are assembled by the tetramerization of TIR domains into two wing regions. Two Ago molecules associate into a symmetric head-to-head dimer, together with the APAZ domain, forming the wing of the butterfly. The two wings, as well as their respective TIR domains, are structurally identical (Supplementary information, Fig. S4c), although they are organized in an asymmetric fashion. Nevertheless, the two TIR-APAZ/Ago units in the same wing adopt different conformations (discussed below) (Fig. 1e; Supplementary information, Fig. S4d), termed A/A’ and B/B’ units, respectively.

### The TIR assembly is indispensable for NADase function

**Fig. 2:**
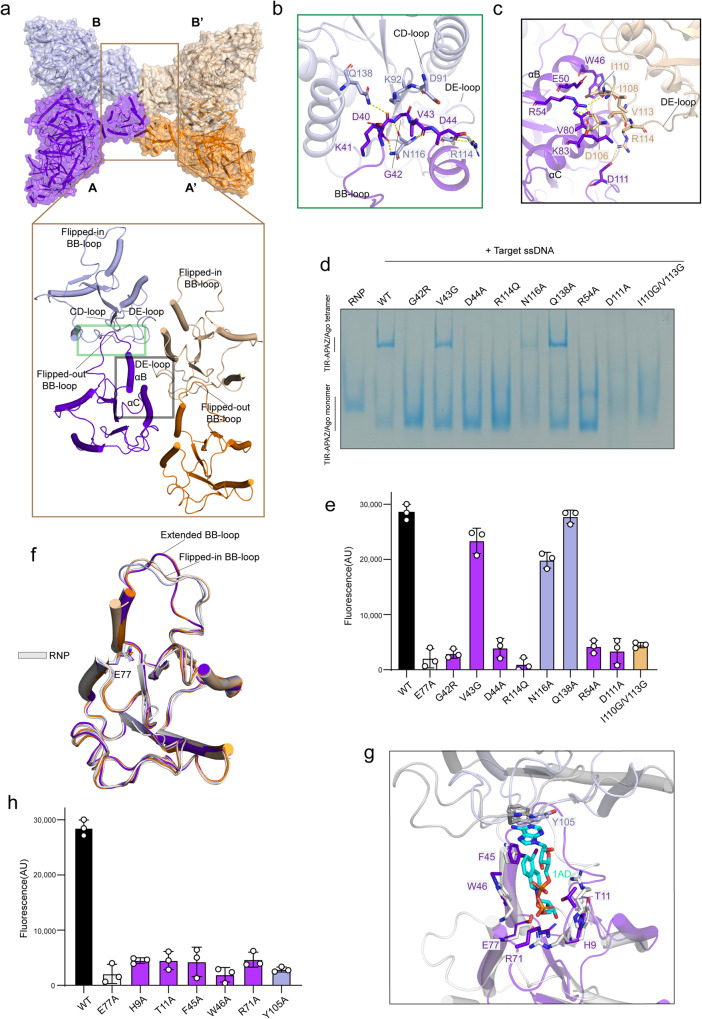
The TIR assembly is essential for NADase activity. **a** The tetramerization of TIR domains. The four TIR-APAZ/Ago units (A/A’/B/B’) are colored in purple, orange, slate, and wheat, respectively. The lower panel shows the intrastrand (green box) and interstrand (black box) contacts formed by TIR domains. The flipped-out BB-loops (purple and orange) may indicate an active state. **b** Detailed insights into the intrastrand interactions. Key interacting residues are shown in stick representation. **c** Detailed insights into the interstrand interactions. Key interacting residues are shown in stick representation. **d** Native PAGE of the wild-type (WT) and mutant TIR-APAZ/Ago complexes. The TIR-APAZ/Ago complex could form a higher-order assembly in the presence of target ssDNA compared to the TIR-APAZ/Ago–gRNA RNP complex. Key residues in the TIR assembly interface were mutated. **e** NAD^+^ hydrolysis by WT or mutant TIR-APAZ/Ago complexes. Mutations of the residues in the TIR assembly interface decreased NAD^+^ cleavage. The E77A catalytic mutant was used as a control. ɛ-NAD^+^ was used as the substrate. Fluorescence intensity (410 nm/310 nm) was measured. The columns are colored the same as the corresponding residues in **b** and **c**. All assays were performed in triplicate, and error bars represent the standard deviations. **f** Superposition of TIR domains in the four units of target DNA-bound TIR-APAZ/Ago complex (the same color scheme as in **a** is used) and in the TIR-APAZ/Ago–gRNA RNP complex (white). **g** Structural comparison of the TIR domains (purple and slate) in TIR-APAZ/Ago–gRNA–ssDNA quaternary complex and the 1AD-bound TIR domains (white and gray) in human SARM1 (PDB ID: 7NAK). 1AD (cyan), the inhibitor of SARM1, has a chemical structure similar to NAD^+^. Residues in the substrate-binding pocket are shown in stick representation. **h** In vitro NAD^+^ degradation assays of WT or mutant TIR-APAZ/Ago protein complexes. Mutations of the residues in the putative substrate-binding pocket substantially weakened or abolished NAD^+^ consumption. The columns are colored the same as the corresponding residues in **g**. All assays were performed in triplicate, and error bars represent the standard deviations.

The TIR domain is usually thought to exert NADase activity through self-association.^15^ Based on analytical ultracentrifugation analysis, the TIR domain-only protein behaved predominantly as a monomer in solution (Supplementary information, Fig. S5a), indicative of an inactive state. As expected, we did not observe obvious NADase activity with TIR domain-only proteins in the fluorescent ε-NAD assay, unless a high concentration of proteins was used (Supplementary information, Fig. S5b). By contrast, TIR-APAZ/Ago complex could efficiently hydrolyze NAD^+^ in the presence of gRNA–DNA duplex. In the target ssDNA-bound complex, four TIR domains are arranged in a parallel two-stranded manner, with each strand consisting of two head-to-tail stacked TIR protomers (Fig. 2a). However, two antiparallel strands of TIR domains are observed in SARM1 and plant TIR domain proteins.^14,19^ Two types of interactions, the intrastrand and interstrand contacts, mediate the formation of the TIR tetramer holoenzyme. In particular, the βB-αB loop (BB-loop) of TIR, which is sandwiched between the βD-βE loop (DE-loop) and the αC-βD loop (CD-loop) of the neighboring TIR, is mainly responsible for the intrastrand association (Fig. 2b). The interstrand interactions are also primarily mediated by the DE-loop, which is bracketed by the αB and αC helices of the adjacent TIR (Fig. 2c). Notably, Arg114 situated in the center of the TIR tetramer, is involved in both intrastrand and interstrand contacts (Supplementary information, Fig. S5c), suggesting a crucial role of Arg114 in TIR self-association. Both polar and hydrophobic interactions appear to be important for the association. Indeed, mutations of either polar or hydrophobic residues in the association interface impaired the TIR-APAZ/Ago oligomerization (Fig. 2d). We next sought to assess the effect of TIR assembly on NADase activity. The mutations that abrogated the higher-order oligomers of TIR-APAZ/Ago, including G42R, D44A and R114Q in the intrastrand interface and R54A, D111A and I110G/V113G in the interstrand interface, substantially weakened or abolished the NAD^+^ consumption by TIR-APAZ/Ago complex (Fig. 2e). These results demonstrate that TIR assembly is required for NADase activity in the TIR-APAZ/Ago system, reminiscent of the TIR-containing proteins in eukaryotic and other prokaryotic immune systems,^14,15,19,21,23,28^ indicating a fundamentally conserved mechanism of TIR activation in the immune response. Surprisingly, it appears that the assembly of the butterfly-shaped TIR-APAZ/Ago complex is maintained mainly by the TIR–TIR interactions, while the oligomerization of other TIR domain proteins is more dependent on oligomerizing domains, such as the NBS-LRR domain in RPP1 and the SAM domain in SARM1.^19,28,37–39^

### The BB-loop potentially modulates substrate recognition

Structural alignment revealed that the four TIR domains in TIR-APAZ/Ago–gRNA–DNA complex are almost identical (Fig. 2f), except for the BB-loop regions. In the A/A’ units, the BB-loop adopts a stretched conformation with regard to the putative active pocket (Fig. 2f; Supplementary information, Fig. S5d). Nonetheless, a flipped-in BB-loop with more flexibility is present in the B/B’ units, which is similar to the BB-loop in RNP complex structure (Fig. 2f; Supplementary information, Fig. S5d). It has been suggested that an ordered BB-loop is required for the NADase function among TIR members.^15^ The highly dynamic BB-loop is possibly bent over the catalytic pocket, therefore at least partially preventing access to the substrate. Likewise, the conformational switch of the BB-loop may modulate the NADase activity in TIR-APAZ/Ago system. As mentioned above, the intrastrand contacts are primarily mediated by the BB-loop, and mutations in BB-loop nearly abolished NAD^+^ hydrolysis (Fig. 2e), further highlighting the importance of the BB-loop in TIR enzymatic activity. Thus, the TIR-APAZ/Ago tetramer may contain two potentially catalytically competent units (A and A’).

Despite extensive trials, we failed to obtain the NAD^+^-bound structure, possibly due to the weak binding that has also been observed in other TIR proteins, such as human SARM1. However, a set of potential active-site residues were revealed through structural comparison with the structural homologs^28^ (Fig. 2g). Specifically, two aromatic residues in αB helix (Phe45 and Trp46) along with Thr11 in βA-αA loop are expected to stack with the nicotinamide moiety of NAD^+^ (Fig. 2g). Alanine substitution of these residues impaired the hydrolysis of NAD^+^, consistent with the proposed model of substrate recognition (Fig. 2h). Interestingly, another aromatic residue, Tyr105, in the adjacent TIR molecule from the same strand is likely to orientate the adenine ring in the suggested model. As anticipated, the Y105A mutation impeded NAD^+^ cleavage (Fig. 2h). Given that Tyr105 does not participate in intrastrand or interstrand association, it is plausible that Tyr105 may be implicated in substrate recognition, further explaining why TIR self-assembly is essential for NADase function. However, further structural study of the NAD^+^-bound complex is needed to validate the proposed mechanism.

### Conformational change of the bridging helix facilitates TIR-APAZ/Ago tetramer assembly

**Fig. 3:**
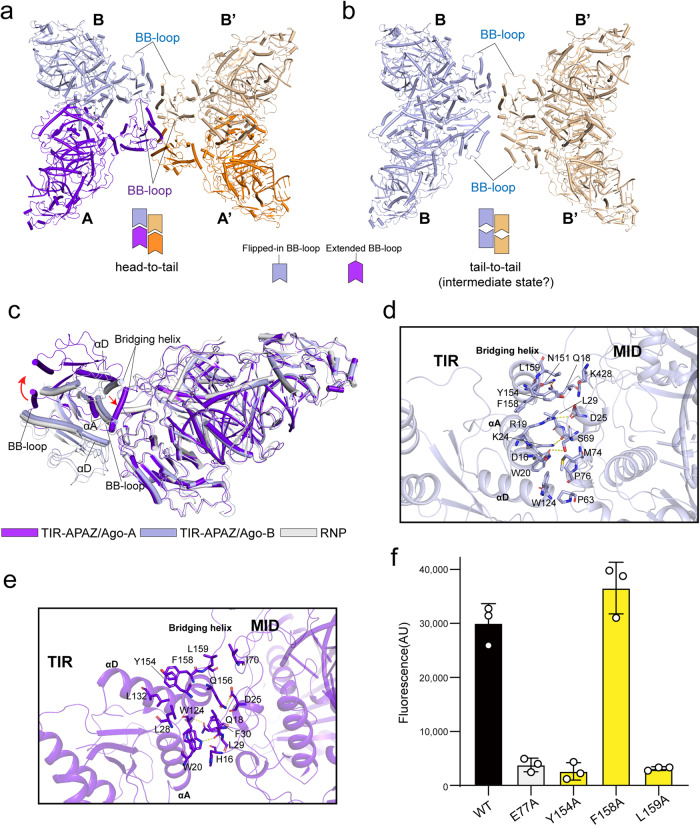
Assembly of TIR-APAZ/Ago complexes is facilitated by the conformational change of the bridging helix. **a** The TIR domains in each strand stack in a head-to-tail manner. The BB-loops are indicated. The same color scheme as in Fig. 2a is used. **b** The TIR domains in the same strand would dock in a tail-to-tail fashion if the active A/A’ units are replaced with the corresponding inactive B/B’ units, indicative of an intermediate state. Therefore, the TIR domains in the two active TIR-APAZ/Ago units (A/A’) would rotate ~180° for the formation of the tetramer. **c** Superposition of the TIR-APAZ molecule in the active unit (A) with those in the inactive unit (B) and TIR-APAZ/Ago–gRNA RNP complex. Compared to the TIR domains in B unit and RNP structure, a clockwise rotation (indicated by red arrows) is observed in the TIR domain of A unit. **d** Close-up view of the interactions between the TIR domain and MID domain in the catalytically inactive TIR-APAZ/Ago unit (B). Key interacting residues are shown in stick representation. **e** Close-up view of the interactions between the TIR domain and MID domain in the catalytically active TIR-APAZ/Ago unit (A). Key interacting residues are shown in stick representation. **f** In vitro NAD^+^ degradation assays of WT or mutant TIR-APAZ/Ago complexes. Mutations of the key residues (Tyr154, Leu159) in the bridging helix suppressed the NAD^+^ cleavage. All assays were performed in triplicate, and error bars represent the standard deviations.

The central TIR assembly would adopt a tail-to-tail arrangement with the BB-loops from the two TIR molecules in each strand exposed in opposite directions, when the active TIR-APAZ/Ago units (A/A’) are replaced with the corresponding inactive TIR-APAZ/Ago units (B/B’) (Fig. 3a, b). Therefore, a conformational rotation should occur to allow the BB-loop (head) to be inserted into the “tail” of the adjacent molecule. As expected, although the TIR or APAZ domain alone superposes well with each other in the quaternary complex (Fig. 2f; Supplementary information, Fig. S5e), massive conformational differences are observed when aligning the whole TIR-APAZ molecules (Supplementary information, Fig. S5f). Specifically, the TIR domains in A/A’ units undergo a clockwise rotation compared to those in B/B’ units (Fig. 3c). However, the overall architecture of the inactive TIR-APAZ molecule aligns well with that in the RNP structure (Fig. 3c), suggesting that target ssDNA binding induces the conformational changes in TIR-APAZ protein to elicit NADase activity. In the B/B’ units, the αA and αD helices of the TIR domain contribute to extensive interactions with the MID domain (Fig. 3d). However, only a few TIR–MID contacts are observed in A/A’ units as the bridging helix relocates to a position between the TIR and MID domains (Fig. 3e), leading to the separation of these two domains. The bridging helix is found to be involved in interactions with the MID domain in both states of TIR-APAZ/Ago units (Fig. 3c–e), albeit at distinct regions. Remarkably, this bridging helix is anchored to the MID domain mainly through the aromatic residue Tyr154 in the B/B’ units (Fig. 3d), which participates in contacts with the TIR domain in the A/A’ units (Fig. 3e). The Y154A mutation decreased NAD^+^ cleavage (Fig. 3f), confirming the importance of the bridging helix in regulating TIR-APAZ/Ago function. Together, it is plausible that the bridging helix might act as a trigger for the tetramerization of the TIR-APAZ/Ago–gRNA–DNA complex.

### Target ssDNA binding induces dimerization of Ago proteins

**Fig. 4:**
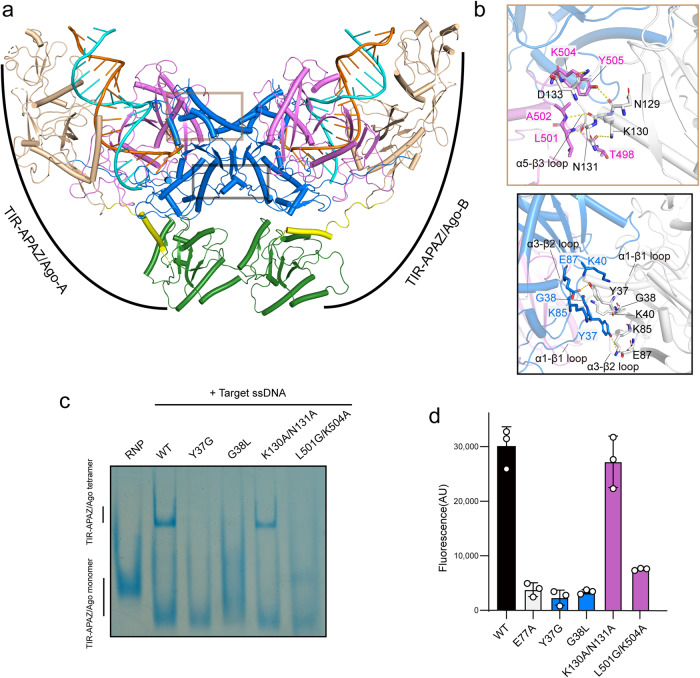
Ago proteins dimerize upon target ssDNA binding. **a** Two Ago molecules in TIR-APAZ/Ago–gRNA–DNA complex interact with each other via the MID and PIWI domains and are organized in a two-fold symmetric configuration. The same color scheme as in Fig. 1b is adopted. **b** Close-up view of the dimerization interface of Ago proteins. Ago protein in the catalytically inactive B unit is depicted in white for clarity. Key interacting residues are shown in stick representation. **c** Native PAGE of WT or mutant TIR-APAZ/Ago complexes. Key residues in the dimerization interface of Ago proteins were mutated. The results suggest that mutations of the residues in the dimerization interface prevent the higher-order assembly of TIR-APAZ/Ago complex. **d** Mutations of the residues in the dimerization interface impaired the NAD^+^ degradation. The columns are colored the same as the corresponding residues in **b**. All assays were performed in triplicate, and error bars represent the standard deviations.

In TIR-APAZ/Ago tetramer complex, two Ago molecules dock onto each other via the MID domain and the C-terminus of the PIWI domain. Close inspection of the structure showed that these two Ago molecules are organized in a two-fold symmetric configuration (Fig. 4a), with the α1–β1 and α3–β2 loops in one molecule packing face-to-face against the equivalent loops in the other molecule and the α5–β3 loop buttressed by the C-terminal loop of PIWI domain from the other molecule (Fig. 4b). The target-induced dimerization of Ago proteins has not been previously reported, which may have a functional role in NADase activation. Indeed, mutation of the residues in the dimerization interface compromised oligomerization (Fig. 4c), and dramatically reduced NAD^+^ degradation (Fig. 4d), implying that the NADase function of TIR domain is dependent on the dimerization of Ago proteins.

Intriguingly, the C-terminal residue (Ile507) of the PIWI domain is also found to coordinate a metal ion, which in turn stabilizes the 5′-phosphate group of gRNA (Supplementary information, Fig. S6a). It has been demonstrated that the 5′-phosphate is crucial for NADase activity.^30^ In the present structure, the 1U nucleotide flips into a pocket between the MID and PIWI domains. The hydrolysis of NAD^+^ was prevented when the residues that orientate the 5′-phosphate moiety were mutated (Supplementary information, Fig. S6b), indicating that the metal-dependent 5′-phosphate recognition is important for NADase activity, akin to the preference of PIWI-clade Ago proteins for 5′-phosphrylated over 5′-hydroxylated guides for target binding and cleavage.^34,35^

### A sensor loop in PIWI domain monitors the formation of gRNA–target ssDNA duplex

**Fig. 5:**
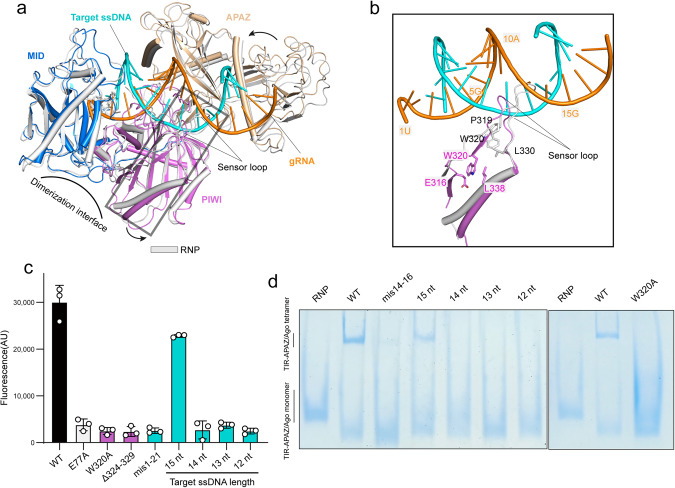
A sensor loop in the PIWI domain probes the complementarity between gRNA and target ssDNA. **a** Structural comparison of Ago proteins in the target-free (RNP, white) and target-bound states (colored by domains). The curved arrow indicates the outward rotation of the PIWI domain after target ssDNA binding. **b** Close-up view of the conformational change of the sensor loop in PIWI domain upon target ssDNA binding. Key residues in the sensor loops are shown in stick representation. **c** In vitro NAD^+^ degradation assays of WT or mutant TIR-APAZ/Ago complexes in the presence of various target ssDNA molecules. 15-nt ssDNA was sufficient to stimulate NAD^+^ cleavage, whereas 14-nt ssDNA failed to activate NADase activity. The columns are colored the same as the corresponding residues in **b**. All assays were performed in triplicate, and error bars represent the standard deviations. **d** Native PAGE of WT or a mutant (W320A) TIR-APAZ/Ago complex in the presence of varied target ssDNA molecules. Target ssDNA shorter than 15 nt was insufficient to induce the higher-order assembly of TIR-APAZ/Ago complex.

Structural comparison of the Ago proteins in the target-free and target-bound states revealed that the overall structures are similar, with subtle differences mainly occurring in the PIWI domain (Fig. 5a). In detail, the PIWI domain rotates away from the MID domain upon target ssDNA binding (Fig. 5a), thus potentially making space for dimerization. Indeed, the two Ago molecules in the target-free state would sterically clash with each other when fixed in the dimerization mode of the target-bound state (Supplementary information, Fig. S7a). Therefore, our structures presumably explain why target ssDNA binding initiates the dimerization of Ago proteins. It is intriguing to note that a long loop (aa 314–332) protruding from the PIWI domain in the target-free complex would pose steric barriers to the propagation of the gRNA–DNA duplex at ~13–15 bp positions (Fig. 5a, b). Our mismatch experiments showed that NAD^+^ cleavage was significantly compromised in the presence of the target ssDNA bearing the mismatch at the central region (14’–16’ nt) (Supplementary information, Fig. S7b), consistent with the previous study.^30^ In addition, the target ssDNA shorter than 15 nt failed to efficiently activate TIR-APAZ/Ago and reduced the formation of TIR-APAZ/Ago tetramer (Fig. 5c, d), suggesting that both faithful pairing at the central region and sufficient duplex length are required to trigger catalysis.

Upon target ssDNA binding, the aforementioned loop (aa 314–332) shifts away from the gRNA–DNA duplex with a stretch of residues (aa 320–322) refolding into β-strand (Fig. 5b), suggesting that this loop may act as a sensor to detect the formation of the gRNA–DNA duplex. This loop is therefore termed as the “sensor loop”. Importantly, deletion of the sensor loop reduced NADase activity (Fig. 5c), supporting the notion that this sensor loop is critical for TIR-APAZ/Ago activation. Trp320 forms a number of contacts within the sensor loop in both states (Fig. 4b), possibly stabilizing the sensor loop. Alanine substitution of Trp320 was found to inhibit TIR-APAZ/Ago activation (Fig. 5c, d), further reinforcing the importance of this sensor loop.

In contrast to the outward movement of PIWI domain, the APAZ domain swings slightly close to the gRNA–DNA duplex (Fig. 5a). A positively charged helix in the APAZ domain inserts into the minor groove of the gRNA–DNA hybrid (Supplementary information, Fig. S7c). However, deletion of this helix had little effect on NAD^+^ cleavage (Supplementary information, Fig. S7d).

### Cryo-EM structure of the target ssDNA-bound SIR2-APAZ/Ago–gRNA quaternary complex

**Fig. 6:**
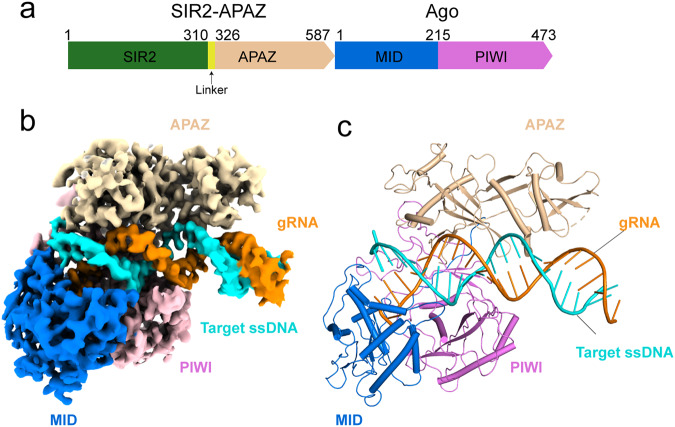
Overall structure of the SIR2-APAZ/Ago complex in target ssDNA-bound state. **a** Domain organization of the SIR2-APAZ and Ago proteins. The SIR2 domain, linker region, APAZ domain, MID domain and PIWI domain are colored in green, yellow, wheat, blue and pink, respectively. **b** Cryo-EM density map of the target ssDNA-bound SIR2-APAZ/Ago complex. The densities of domains are colored as in **a**. The SIR2 domain is invisible due to flexibility. The gRNA is colored in orange and the target ssDNA is shown in cyan. **c** Atomic model of the target ssDNA-bound SIR2-APAZ/Ago complex. The structure is shown in cartoon representation with the same color scheme as in **b**.

SIR2-APAZ is also reported to form a heterodimeric complex with the Ago protein encoded in the same operon^31^ (Fig. 6a). Akin to the TIR-APAZ/Ago system, target ssDNA binding could trigger the NADase activity of SIR2-APAZ/Ago system (Supplementary information, Fig. S8a, b). Nonetheless, the SIR2-APAZ/Ago complex does not assemble into higher-order oligomers in the presence of gRNA–DNA duplex (Supplementary information, Fig. S8c), in contrast to the higher-order oligomerization of the TIR-APAZ/Ago complex. To explore the molecular basis of SIR2-APAZ/Ago system, we determined the cryo-EM structure of the target ssDNA-bound quaternary complex from *Geobacter sulfurreducens* at a resolution of 3.0 Å (Fig. 6b, c; Supplementary information, Fig. S9 and Table S1). The Ago protein and the APAZ domain of SIR2-APAZ, as well as a ~25-bp gRNA–DNA hybrid, could be clearly assigned in the present EM map (Supplementary information, Fig. S10a). However, the SIR2 domain is missing, as discussed below.

The resolved complex adopts a canonical bilobed Ago architecture, with the gRNA–DNA duplex inserted through the central channel formed by Ago protein and the APAZ domain of SIR2-APAZ (Supplementary information, Fig. S10a). The flipped 1A of gRNA is sandwiched between Phe158 and His166 (Supplementary information, Fig. S10b), with the 5′-phosphate group recognized in a conserved metal-dependent fashion, similar to that in TIR-APAZ/Ago system. Mutation of either Phe158 or His166 had little impact on NAD^+^ degradation (Supplementary information, Fig. S10c). Surprisingly, Arg190 protruding from MID domain orientates the base group of 2U (Supplementary information, Fig. S10d), thus blocking the potential base pairing between 2U and 2’dA. As a result, the 2’dA of target ssDNA flips out and projects into a pocket formed by Ago protein and the APAZ domain (Supplementary information, Fig. S10d). However, deletion of 1’dT–2’dA displayed only a mild effect on NAD^+^ hydrolysis (Supplementary information, Fig. S10e). Likewise, the hydrolysis of NAD^+^ was not affected when the residues located in the 2’dA-binding pocket were mutated (Supplementary information, Fig. S10f).

### Target ssDNA binding unleashes the NADase activity of SIR2 domain

The apo SIR2-APAZ/Ago structure predicted by AlphaFold with high confidence (termed AF structure),^36^ which is consistent with the previous SAXS study,^31^ was used to explore the activation mechanism. In the AF structure, the Ago protein is encircled by the separated SIR2 and APAZ domains of SIR2-APAZ protein (Supplementary information, Fig. S11a). The predicted SIR2 domain of SIR2-APAZ/Ago system, which is expected to primarily interact with the PIWI domain, is structurally similar to that of the ThsA effector in the Thoeris antiphage defense system^27,40^ (Supplementary information, Fig. S11b, c). Destabilization of the SIR2 domain is suggested to be required for the NADase activity of ThsA, which is in line with our observation that the SIR2 domain is dissociated from Ago protein and is not visible in the active SIR2-APAZ/Ago–gRNA–DNA complex. A helical subdomain, which is located above the catalytic pocket of the SIR2 domain, is reported to regulate NADase activity through a closed-to-open transition in ThsA^27^ (Supplementary information, Fig. S11b, c). A similar scenario may also apply to the SIR2-APAZ/Ago system. The equivalent helical subdomain (“lid”) seems to adopt a closed configuration that blocks substrate access to the active site in the AF structure. As anticipated, replacement of the region (aa 83–94) covering the catalytic pocket with a flexible linker (GSAGSAG) restored the NADase activity of the SIR2 domain-only protein (Supplementary information, Fig. S11d).

**Fig. 7:**
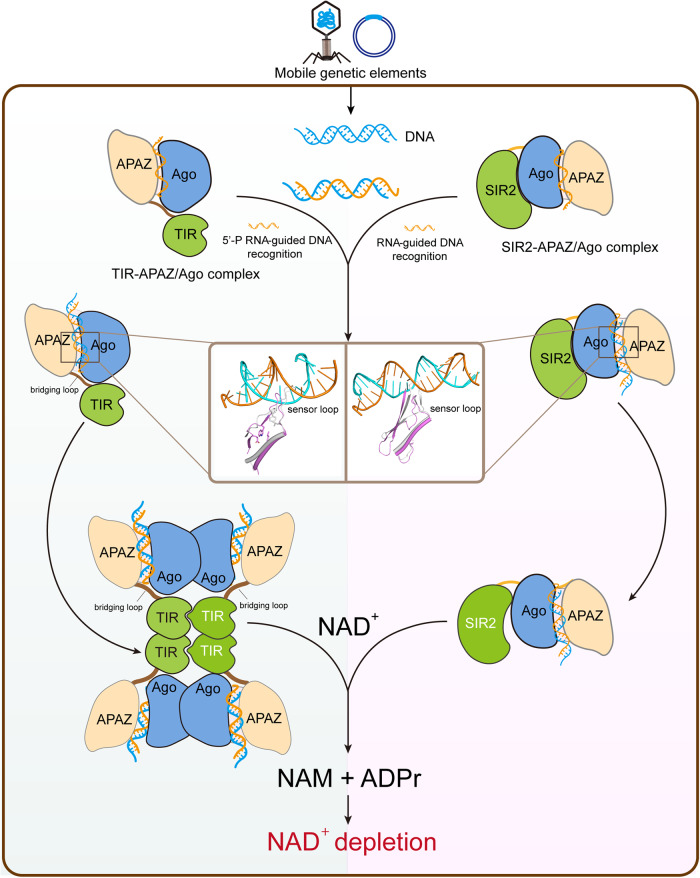
Schematic diagram illustrating the NADase activation mechanisms of the TIR-APAZ/Ago and SIR2-APAZ/Ago systems. Both systems utilize a conserved sensor loop in the PIWI domain of Ago protein to detect the formation of the gRNA–target ssDNA duplex, thereby initiating the conformational changes required for the activation of the NADase activity in TIR domain or SIR2 domain. While TIR domain oligomerization is needed to form the active pocket in TIR-APAZ/Ago system, the target ssDNA binding activates SIR2 domain by increasing flexibility.

Interestingly, a long loop (aa 261–276, termed sensor loop) in PIWI domain shifts away from the gRNA–DNA duplex compared to that in the AF structure (Supplementary information, Fig. S12a, b). In AF structure, the sensor loop would overlap with the gRNA–DNA duplex at ~13–15 bp positions (Supplementary information, Fig. S12b), suggesting that this loop may inspect the base pairing between gRNA and target ssDNA. The NAD^+^ cleavage was almost blocked when the sensor loop was deleted (Supplementary information, Fig. S12c). Furthermore, deletion and mismatch experiments showed that the imperfect pairing at 13–15 bp impaired NADase function (Supplementary information, Fig. S12c, d). Notably, this sensor loop is spatially and functionally equivalent to the sensor loop in TIR-APAZ/Ago system, suggesting a conserved mechanism for target recognition and activation of NADase activity in pAgo-associated NADase systems (Fig. 7). However, further structural study of SIR2-APAZ/Ago complex in the absence of target ssDNA is needed to discern the role of this sensor loop in modulating NADase activity.

## Discussion

There is emerging evidence indicating that depletion of NAD^+^ is a shared cell death signaling pathway among eukaryotic and prokaryotic immune systems. In the bacterial anti-mobile element systems, the short pAgos were found to be associated with proteins containing NADase domains which compensate for the loss of nuclease activity in the PIWI domain.^30,31^ Here, we structurally and biochemically characterized two pAgo-associated NADase systems (TIR-APAZ/Ago and SIR2-APAZ/Ago), revealing both diverse and conserved mechanisms of NADase activation in bacterial immune response.

The binding of gRNA–DNA heteroduplex is a common mechanism adopted by these two systems to elicit NADase activity. The TIR-mediated NADase activity is unleashed by the gRNA–DNA heteroduplex-triggered assembly of the TIR-APAZ/Ago tetramer. However, the engagement of the gRNA–DNA hybrid does not alter the oligomerization state of the SIR2-APAZ/Ago complex (Supplementary information, Fig. S8c). The target ssDNA binding induces the dimerization of Ago proteins via the outward movement of the PIWI domain, possibly facilitating the tetramerization of the TIR-APAZ/Ago–gRNA–DNA complex. Although the target ssDNA binding is expected to trigger the conformational change of PIWI domain in SIR2-APAZ/Ago system, the corresponding dimerization interface is occupied by SIR2 domain, partially explaining why the SIR2-APAZ/Ago complex does not form higher-order oligomers. We identified a functionally conserved loop (sensor loop) in PIWI domain that senses the gRNA–DNA hybrid in both TIR-APAZ/Ago and SIR2-APAZ/Ago systems. It is conceivable that the sensor loop recognizes the base pairing at ~15th position of gRNA, and functions as a molecular checkpoint to stimulate the conformational changes of PIWI domain to facilitate catalytic reaction. Interestingly, this loop is equivalent to the catalytic loop in the canonical active Ago proteins, suggesting that the nuclease function has been switched to control NADase activity by probing base pairing. It is intriguing to speculate that the equivalent loop in the DUF4365/Ago system might be implicated in the regulation of the nuclease function attributed to the DUF4365 domain through the detection of the gRNA–DNA duplex.^41^

Similar to the canonical pAgo proteins, the 5′-phosphate group of gRNA is recognized in a metal-dependent manner for both TIR-APAZ/Ago and SIR2-APAZ/Ago systems. Nonetheless, it appears that the 5′-phosphate moiety is required to activate the TIR-APAZ/Ago system but not the SIR2-APAZ/Ago system, as supported by the mutagenesis studies of the corresponding binding pocket (Supplementary information, Figs. S6b, S10c). Given that the uracil base of 1U in TIR-APAZ/Ago system is much smaller than the adenine base of 1A in SIR2-APAZ/Ago system, the recognition and stabilization of 1U might be more dependent on the interactions with residues of Ago protein, thus providing an alternative explanation for these mutagenesis results. Several well-characterized Ago proteins, such as *Rhodobacter sphaeroides* Ago (RsAgo)^42^ and human Ago2,^43^ show preference for uridine nucleotides at the 5′-end of gRNA.^6^ Similarly, the TIR-APAZ/Ago system can be activated by 5′-uridine but not 5′-adenosine gRNA (Supplementary information, Fig. S13a), likely due to the relatively small binding pocket for the 5′-end of gRNA. However, both 5′-uridine and 5′-adenosine gRNA can robustly activate the NADase activity of the SIR2-APAZ/Ago system (Supplementary information, Fig. S13b). In contrast to the typical Ago proteins, the nucleotide at the 2’ position of target ssDNA does not base pair with gRNA, and flips into a pocket mainly created by the MID domain in SIR2-APAZ/Ago complex (Supplementary information, Fig. S10d). However, the equivalent binding pocket is absent in TIR-APAZ/Ago complex, explaining why the 3′-end of target ssDNA, such as 1’dA, could not be observed. While the 3′-end of target ssDNA in these two systems adopts different conformations, recognition of the 3′-end is dispensable for the NADase function, as evidenced by the truncation experiments (Supplementary information, Figs. S6b, S10e). Unlike the classical Ago proteins, perfect base pairing between the seed region and the target seems unnecessary for TIR-APAZ/Ago activation (Supplementary information, Fig. S7b, d). Together, the functional APAZ/Ago modules in these two systems display typical bilobed organization, with conserved and divergent features evolved to adapt to immune response.

Depletion of NAD^+^ is widely found to elicit cell death in bacteria–phage arms race.^44^ The two NADase domains, TIR and SIR2, engage Ago proteins through different interfaces (TIR–MID and SIR2–PIWI) in TIR-APAZ/Ago and SIR2-APAZ/Ago systems. While TIR and SIR2 are both activated by the binding of target ssDNA, the detailed mechanisms vary between them. In the resting state, the NADase activity of TIR is likely to be self-inhibited partially by the flexible BB-loop. In the active state, the BB-loop is fixed by an adjacent TIR molecule and exhibits an extended conformation allowing the access of the substrate to the active site. By contrast, the entrance to the catalytic pocket is proposed to be covered by the helical subdomain of SIR2 in the resting state (Supplementary information, Fig. S11b, c), which is potentially held in place by interacting with Ago protein (Supplementary information, Fig. S11a). When activated by target ssDNA, the interaction between the helical subdomain and Ago is assumed to be altered, and the helical subdomain is thought to move away with increased flexibility (Supplementary information, Fig. S12a). While the regulatory elements in these two systems undergo different transitions (disordered-to-ordered for BB-loop and ordered-to-disordered for helical subdomain), the outcomes are the same: the entrance to the active site is opened. For TIR, self-association is essentially required for NADase function, not only because the BB-loop is stretched out by the neighboring TIR domain, but also because the adjacent molecule contributes to the constitution of the active site (such as Tyr105) (Fig. 2g). Intriguingly, since the BB-loop is directly involved in the intrastrand association, a more likely scenario is that the BB-loop functions to facilitate and stabilize the formation of the composite active site from the two TIR molecules in the same strand. However, the active site might be limited to the single SIR2 domain for SIR2-APAZ/Ago system.

In summary, our work elucidates the molecular basis for pAgo-associated NADase function, revealing shared conformational features upon NADase activation mediated by distinct mechanisms. These results also establish a foundation for understanding the short pAgo in bacterial immunity.

## Materials and methods

### Protein expression and purification

For the expression and purification of the TIR-APAZ/Ago system from *M. polysiphoniae*, the genes encoding Ago and TIR-APAZ proteins were synthesized by GENEWIZ. The sequence for Ago protein was cloned into the pET vector containing an N-terminal His-MBP tag, and the gene for TIR-APAZ was cloned into the 13S-A vector (Addgene: 48323). All resultant plasmids were co-transformed into BL21(DE3) cells. Protein expression was induced by 0.2 mM isopropyl β-D-thiogalactopyranoside (IPTG). Cells were harvested and lysed by sonication in a lysis buffer containing 20 mM Tris-HCl, pH 7.5, 400 mM NaCl, and 2 mM β-mercaptoethanol. Ni-NTA resin (Qiagen) was incubated with the clarified cell lysate and extensively washed with the binding buffer. The target protein complex was eluted with an elution buffer containing 25 mM Tris-HCl, pH 7.5, 500 mM NaCl, 300 mM imidazole, and 2 mM β-mercaptoethanol. Home-made TEV was used to remove the N-terminal His-MBP tag. The TIR-APAZ/Ago complex was further purified using a HiTrap Heparin HP column (Cytiva). Peak fractions containing target protein complex were then concentrated and applied to a Superdex 200 column (Cytiva) equilibrated with the gel-filtration buffer 1 (25 mM Tris-HCl, pH 7.5, 150 mM NaCl, 2 mM DTT, and 2 mM MgCl_2_). Peak fractions containing the target protein complex were collected and analyzed by SDS-PAGE.

For the expression and purification of the SIR2-APAZ/Ago system from *G. sulfurreducens*, the genes encoding the Ago and SIR2-APAZ proteins were synthesized by GENEWIZ and cloned into the 13S-A vector (Addgene: 48323) and the pET vector containing an N-terminal His-SUMO tag, respectively. Ago and SIR2-APAZ proteins were co-expressed in BL21(DE3) cells following induction with 0.2 mM IPTG. The target protein complex was then purified using Ni-NTA resin (Qiagen) and the HiTrap Heparin HP column (Cytiva) as described above. Finally, the elution was concentrated and fractionated on a Superdex 200 column (Cytiva) with the gel-filtration buffer 2 (25 mM HEPES, pH 7.9, 150 mM NaCl, 2 mM DTT, and 2 mM MgCl_2_). Peak fractions containing the target protein complex were collected and analyzed by SDS-PAGE.

### ɛ-NAD^+^ degradation assays

ɛ-NAD^+^ (Nicotinamide 1,*N*^6^-ethenoadenine dinucleotide) degradation assays were performed in a reaction buffer containing 10 mM MES, pH 6.8, 150 mM NaCl and 5 mM MgCl_2_. 1 μM TIR-APAZ/Ago or SIR2-APAZ/Ago protein complex was pre-incubated with gRNA at 37 °C for 15 min. Target DNA was then added to the mixture and incubated for 1 h at 37 °C. The molar ratio of TIR-APAZ/Ago complex, gRNA and target DNA in the reaction system was 1:0.3:0.3, and the molar ratio of SIR2-APAZ/Ago complex, gRNA and target DNA in the reaction system was 1:0.7:0.7. After incubation, 50 μM ɛ-NAD^+^ (Sigma-Aldrich) was added to the reaction system. NAD hydrolysis was carried out at 37 °C and monitored using a BioTek Synergy H1 Plate Reader with an excitation wavelength of 310 nm and an emission wavelength of 410 nm. All assays were performed in triplicate, and the standard deviations were calculated with GraphPad Prism v.8.3.

### Native polyacrylamide gel electrophoresis (PAGE)

Purified TIR-APAZ/Ago complex was diluted to 0.2 mg/mL and incubated at 37 °C for 15 min with the gRNA in a 1:1.5 molar ratio. An equal amount of target DNA as gRNA was then added to the mixture and incubated for another 1 h at 37 °C. The samples were separated by 5% native page gel, and the separated protein bands were visualized using Coomassie Brilliant Blue staining.

### Size exclusion chromatography

Size exclusion chromatography was carried out using an AKTA Purifier system (GE Healthcare). Purified TIR-APAZ/Ago complex was incubated with the gRNA in the gel filtration buffer (25 mM Tris-HCl, pH 7.5, 150 mM NaCl, 2 mM DTT, 2 mM MgCl_2_) at 37 °C for 15 min. The DNA target was added to the mixture and incubated for another 1 h. The molar ratio of TIR-APAZ/Ago complex, gRNA, and target DNA in the sample was 1:1.2:1.5. After incubation, the samples were loaded onto a Superdex 200 Increase 10/300 GL column (Cytiva). Peak fractions were collected and analyzed by SDS-PAGE.

### Analytical ultracentrifugation

Purified TIR domain was prepared in the gel filtration buffer and diluted to 1 mg/mL. Sedimentation velocity measurements were carried out using a Beckman Optima XL-I analytical ultracentrifuge. Sedimentation coefficient was analyzed using the Sedfit and Sedphat programs.^45^

### Cryo-EM sample preparation

Purified TIR-APAZ/Ago protein complex was mixed with 5′-phosphorylated gRNA (UGAGGUAGUAGGUUGUAUAGU, Sangon Biotech) alone or together with the target ssDNA (TACTATACAACCTACTACCTCAT, Sangon Biotech) at a molar ratio of 1:1.5:2 for the reconstitution of the TIR-APAZ/Ago–gRNA and TIR-APAZ/Ago–gRNA–DNA complexes. An aliquot of 3.5 μL of the assembled complexes (0.5 mg/mL) was applied to glow-discharged Au R1.2/1.3 holey carbon grid (200 or 300 mesh, Quantifoil). After incubation for 20 s, the grids were blotted for 2 s at 100% humidity and 4 °C, and plunge-frozen in liquid ethane using Vitrobot Mark IV (Thermo Fisher Scientific, Waltham, USA).

For the preparation of the SIR2-APAZ/Ago complexed with gRNA and target ssDNA, SIR2-APAZ/Ago was mixed with the synthesized 5′-phosphorylated gRNA (AUAAUGGUUUCUUAGACGUCGUUUUAGAGCUGUGUUGUUUCG, Sangon Biotech) alone or together with the synthesized target DNA (CGAAACAACACAGCTCTAAAACGACGTCTAAGAAACCATTAT, Sangon Biotech) at a molar ratio of 1:1.5:2. The aliquots of 3.5 μL prepared samples (0.7 mg/mL) were applied to the glow-discharged Quantifoil holey carbon girds (Au, R1.2/1.3, 300 mesh). The grids were blotted with force 2 for 8 s and plunged into liquid ethane using the Vitrobot Mark IV.

### Cryo-EM data collection

Cryo-EM data of TIR-APAZ/Ago–gRNA and the SIR2-APAZ/Ago–gRNA–DNA complexes were collected with a Titan Krios microscope (Thermo Fisher Scientific, Waltham, USA) operated at 300 kV and images were collected using EPU^46^ at a nominal magnification of 105,000× (resulting in a calibrated physical pixel size of 0.85 Å/pixel) with a defocus range of –1.2 μm to –2.2 μm. The images were recorded on a K3 summit electron direct detector in the super-resolution mode at the end of a GIF-Quantum energy filter operated with a slit width of 20 eV. A dose rate of 15 electrons per pixel per second and an exposure time of 2.5 s were used, generating 40 movie frames with a total dose of ~54 e/Å^2^. A total of 2221 and 3589 movie stacks were collected for TIR-APAZ/Ago–gRNA complex and SIR2-APAZ/Ago–gRNA–DNA complex, respectively.

The grids for TIR-APAZ/Ago–gRNA–DNA were loaded into a CRYO ARM 300 electron microscope (JEOL, Japan) operating at 300 kV with a K3 direct electron detector (Gatan, USA). Cryo-EM images were recorded automatically using Serial-EM software^47^ in the super-resolution mode with a super-resolution pixel size of 0.475 Å/pixel at defocus values ranging from –0.5 µm to –2.5 µm at a calibrated magnification of 50,000×. Data were collected at a frame rate of 40 frames per second with a total electron dose of 40 e/Å^2^.

### Cryo-EM image processing

Cryo-EM data of TIR-APAZ/Ago–gRNA were processed using RELION-3.^48^ Movie frames were aligned using MotionCor2^49^ with a binning factor of 2. Contrast transfer function (CTF) parameters were estimated using Gctf.^50^ Around 12,000 particles were auto-picked without template to generate 2D averages for subsequent template-based auto-picking. 4,037,142 particles were auto-picked and extracted from the dose-weighted micrographs. 2D classification was performed to exclude bad particles. 868,883 particles were selected for further processing. Particles from different views were used to generate initial model in cryoSPARC.^51^ 3D classification was performed to distinguish different conformational states. 373,126 particles were used for final homogenous 3D refinement, CTF refinement, and Bayesian polishing, converging at 3.7 Å resolution.

Image processing for the TIR-APAZ/Ago–gRNA–DNA complex was performed with cryoSPARC.^51^ The patch motion correction and CTF estimation of the recorded movies were performed with cryoSPARC.^51^ Particles were automatically selected using Blob picker or Topaz and then extracted from the micrographs with a box size of 300 pixels and subsequently subjected to 2D classification. Good classes were selected with two rounds of ab-initio reconstruction and then heterogeneous refinement. Two predominant classes containing 369,588 particles were selected for non-uniform refinement (NU-refinement), yielding a final reconstruction with an overall resolution of 2.95 Å.

Cryo-EM data of the SIR2-APAZ/Ago–gRNA–DNA complex were processed using cryoSPARC.^51^ From the 3352 motion-corrected and dose-weighted micrographs, 2,890,897 particles were picked using Blob picker and extracted at a pixel size of 0.9067 Å. 2D classification was performed to remove bad particles, and 972,336 particles were selected for subsequent processing. Ab-initio reconstruction and heterogeneous refinement were performed to select classes containing homogenous particles. The best class containing 380,948 particles were subjected to the homogeneous refinement and the NU-refinement, yielding a final reconstruction at 3.0 Å resolution.

### Model building and refinement

Cryo-EM maps were sharpened by phenix.auto_sharpen.^52–54^ Atomic models of the TIR-APAZ/Ago and SIR2-APAZ/Ago protein complexes predicted by AlphaFold^36^ were used as the initial templates. The initial models were adjusted and refined manually in Coot.^55^ Further real-space refinements were carried out using phenix.real_space_refine.^56^ The models of gRNA and target DNA were built manually in Coot and iteratively refined with phenix.real_space_refine. The protein complexes and nucleic acid models were then combined for further refinements in Phenix. The quality of the models was analyzed using MolProbity^57^ in Phenix. Refinement statistics are summarized in Supplementary information, Table S1.

## Supplementary information

Supplementary information, Fig. S1

Supplementary information, Fig. S2

Supplementary information, Fig. S3

Supplementary information, Fig. S4

Supplementary information, Fig. S5

Supplementary information, Fig. S6

Supplementary information, Fig. S7

Supplementary information, Fig. S8

Supplementary information, Fig. S9

Supplementary information, Fig. S10

Supplementary information, Fig. S11

Supplementary information, Fig. S12

Supplementary information, Fig. S13

Supplementary information, Table S1

## Data Availability

The atomic coordinates and EM maps have been deposited in the Protein Data Bank and the Electron Microscopy Data Bank, respectively, under accession codes 8I87/EMD-35240 (TIR-APAZ/Ago–gRNA–DNA), 8I88/EMD-35241 (TIR-APAZ/Ago–gRNA) and 8IN8/EMD-35592 (SIR2-APAZ/Ago–gRNA–DNA).
