# Supplementary material for: Structural insights into mechanisms of Argonaute protein-associated NADase activation in bacterial immunity
Source: Cell Res. 2023 Jun 13;33(9):699–711. doi: 10.1038/s41422-023-00839-7 (PMC10474274; doi:10.1038/s41422-023-00839-7)
Supplement: Supplementary file 1 — Supplementary information, Fig. S1 [file 41422_2023_839_MOESM1_ESM.pdf]

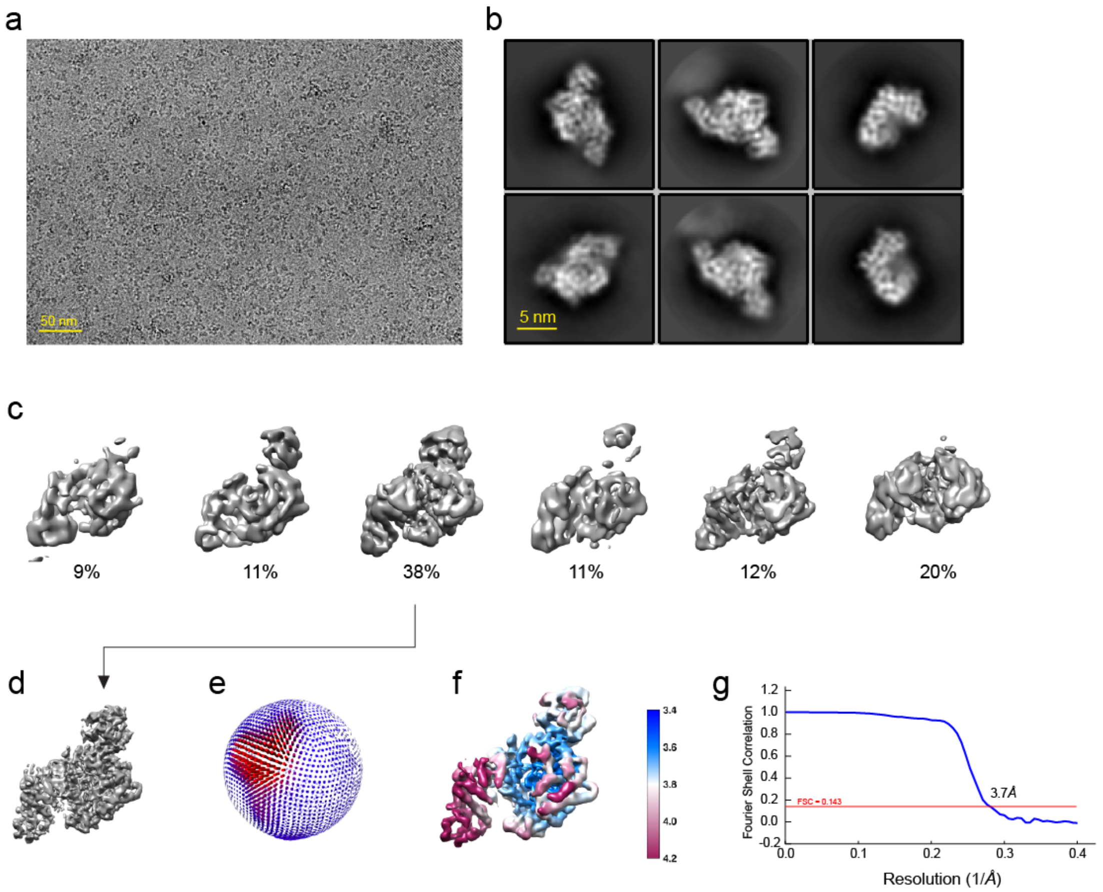

Supplementary information Figure S1. Cryo-EM reconstruction of the TIR-APAZ/Ago in complex with gRNA. a, A representative cryo-EM micrograph. **b,** Representative 2D class averages. **c,** 3D classes resolved in 3D classification. **d,** Final map of TIR-APAZ/Ago. **e,** View distribution of the reconstruction. **f,** Local resolution analysis of the reconstructed map. **g,** The Fourier Shell Correlation (FSC) curve of the reconstruction. The resolution of the reconstruction was estimated using the gold-standard cutoff of FSC=0.143.
