# Supplementary material for: Structural insights into mechanisms of Argonaute protein-associated NADase activation in bacterial immunity
Source: Cell Res. 2023 Jun 13;33(9):699–711. doi: 10.1038/s41422-023-00839-7 (PMC10474274; doi:10.1038/s41422-023-00839-7)
Supplement: Supplementary file 2 — Supplementary information, Fig. S2 [file 41422_2023_839_MOESM2_ESM.pdf]

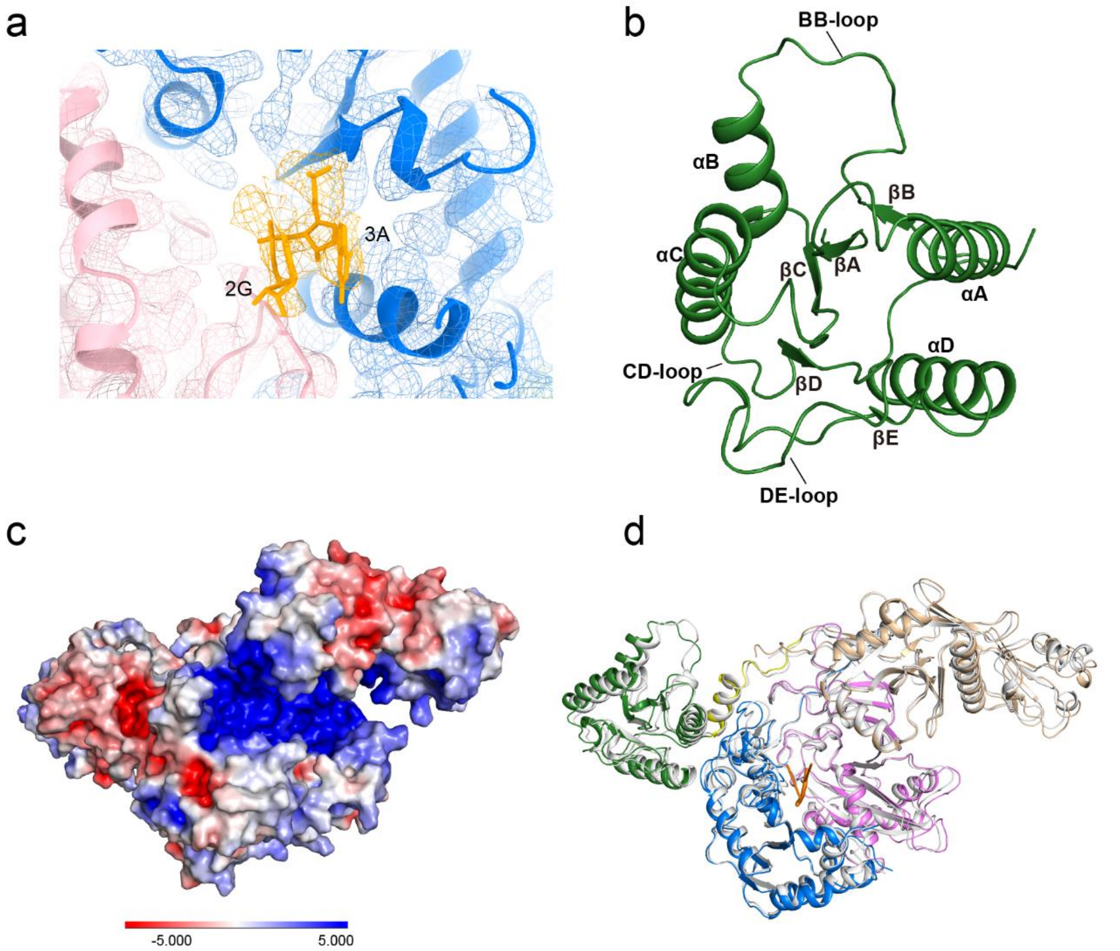

Supplementary information Figure S2. Structural features of the gRNA-bound TIR-APAZ/Ago complex. a, Cryo-EM density (orange mesh) and the atomic model (orange sticks) of the seed region in the gRNA. **b,** Overall structure of the TIR domain in the TIR-APAZ/Ago complex. **c,** Electrostatic surface representations of the TIR-APAZ/Ago complex. Ago protein and the APAZ domain form a positively charged channel. The seed of gRNA is omitted for clarity. **d,** Superposition of the gRNA-bound TIR-APAZ/Ago complex (colored by domains) and the apo TIR-APAZ/Ago model predicted by AlphaFold (white).
