# Supplementary material for: Structural insights into mechanisms of Argonaute protein-associated NADase activation in bacterial immunity
Source: Cell Res. 2023 Jun 13;33(9):699–711. doi: 10.1038/s41422-023-00839-7 (PMC10474274; doi:10.1038/s41422-023-00839-7)
Supplement: Supplementary file 3 — Supplementary information, Fig. S3 [file 41422_2023_839_MOESM3_ESM.pdf]

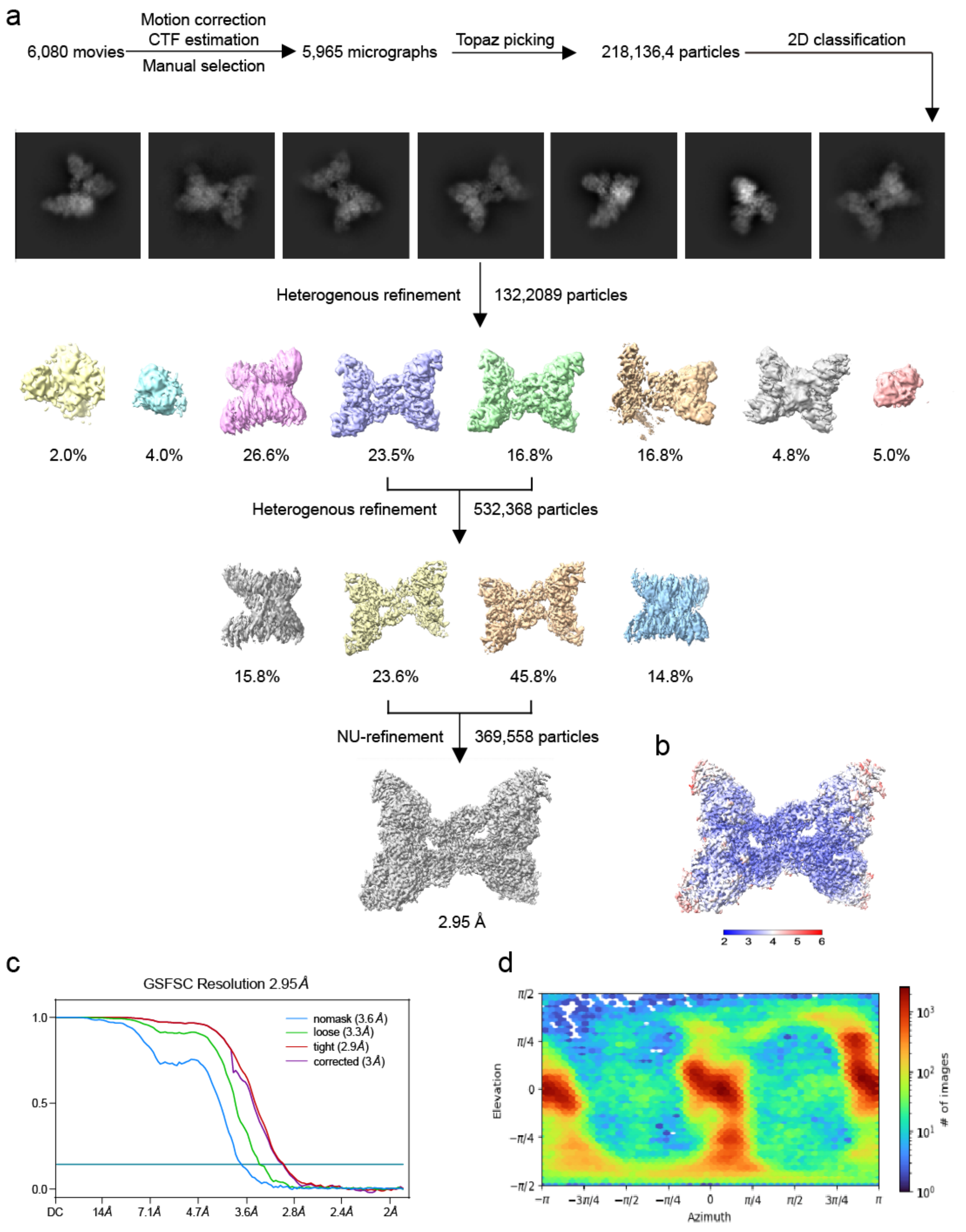

Supplementary information Figure S3. Cryo-EM reconstruction of the TIR-APAZ/Ago-gRNA-DNA complex. a, Flowchart of cryo-EM data processing. **b,** Cryo-EM density map colored by local resolution. **c,** Fourier shell correlation (FSC) curve calculated using the two independent half-maps. The resolution of the reconstruction was estimated using the FSC=0.143 cutoff. **d,** Orientation distribution of the refined particles in the final reconstruction.
