# Supplementary material for: Structural insights into mechanisms of Argonaute protein-associated NADase activation in bacterial immunity
Source: Cell Res. 2023 Jun 13;33(9):699–711. doi: 10.1038/s41422-023-00839-7 (PMC10474274; doi:10.1038/s41422-023-00839-7)
Supplement: Supplementary file 4 — Supplementary information, Fig. S4 [file 41422_2023_839_MOESM4_ESM.pdf]

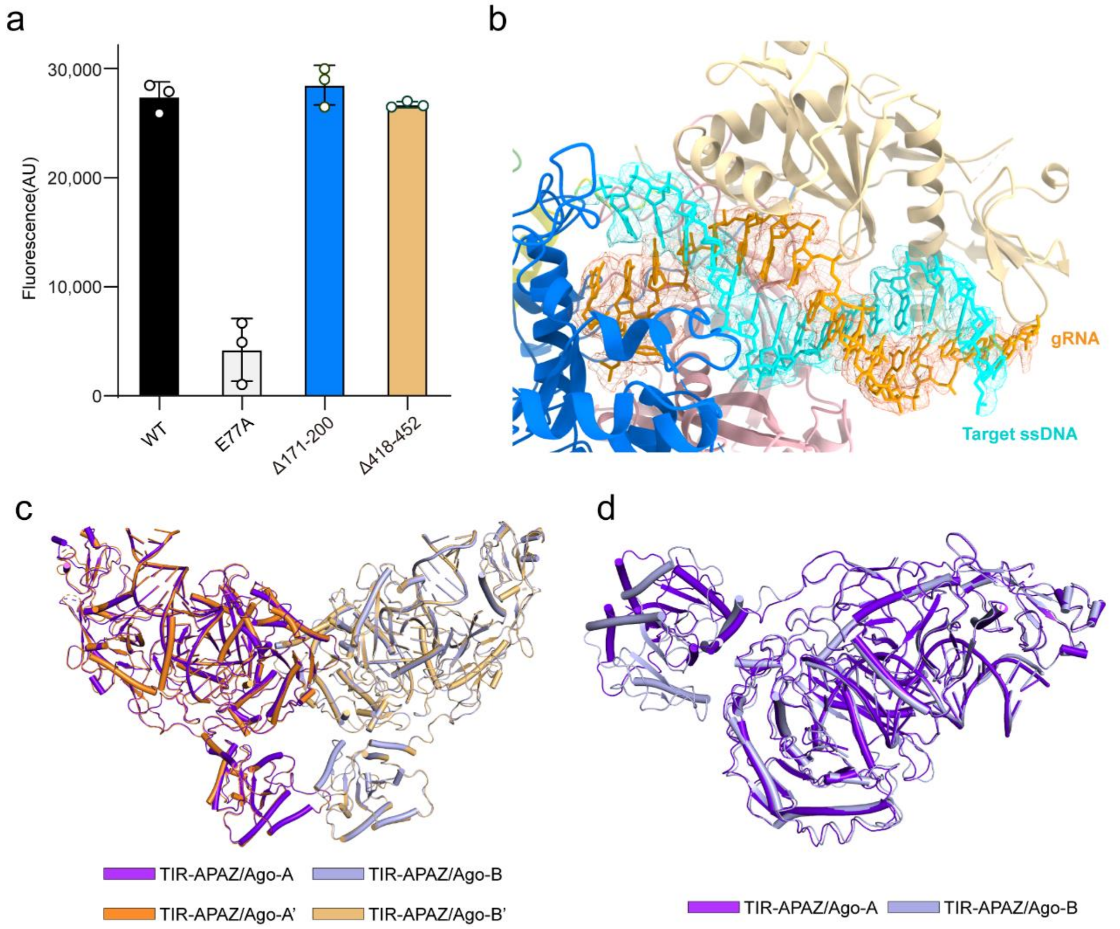

Supplementary information Figure S4. Overall structure of the target ssDNA-bound TIR-APAZ/Ago complex. a, In vitro NAD^+ hydrolysis to investigate the impact of the deletion mutation in the APAZ C-terminal segment and a stretch of amino acids (aa 418-452) in the MID domain on the NADase activity of the TIR-APAZ/Ago system. The E77A catalytic mutant was used as the control. The columns are colored the same as the corresponding residues in Fig. 1a. All assays were performed in triplicate, and error bars represent the standard deviations. **b,** Cryo-EM density and the atomic model of the gRNA (orange) -target ssDNA (cyan) duplex. **c,** Superposition of the two "wing" regions (A/B *vs.* A'/B') in the "butterfly-shaped" TIR-APAZ/Ago-gRNA-DNA tetramer complex. **d,** Superposition of the two distinct types of units (A and B) in the TIR-APAZ/Ago-gRNA-DNA complex.
