# Supplementary material for: Structural insights into mechanisms of Argonaute protein-associated NADase activation in bacterial immunity
Source: Cell Res. 2023 Jun 13;33(9):699–711. doi: 10.1038/s41422-023-00839-7 (PMC10474274; doi:10.1038/s41422-023-00839-7)
Supplement: Supplementary file 5 — Supplementary information, Fig. S5 [file 41422_2023_839_MOESM5_ESM.pdf]

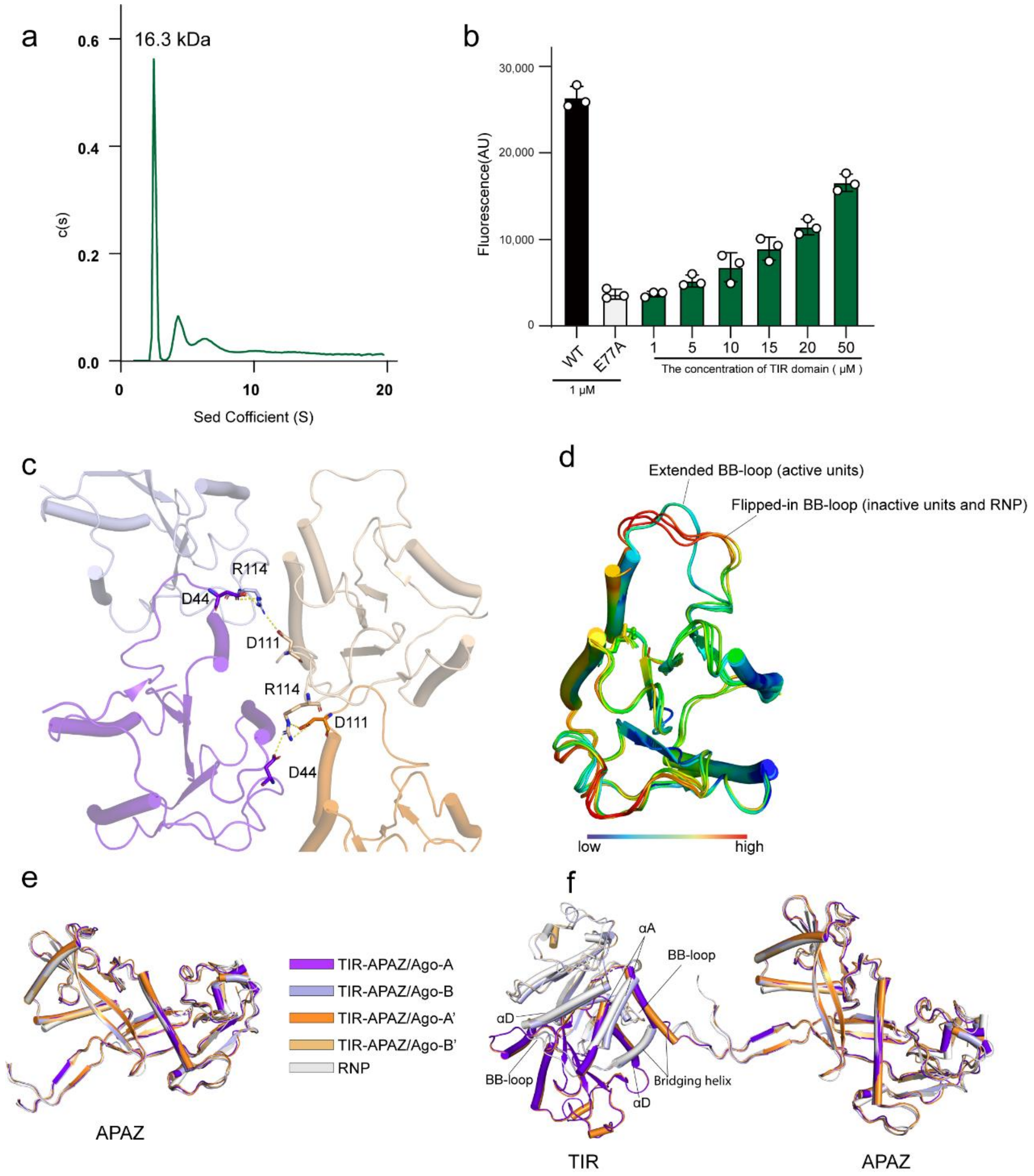

Supplementary information Figure S5. The role of TIR assembly in NADase activation. a, Analytical ultracentrifugation analysis of the TIR domain. For TIR domain-only protein (residues 1-159), the theoretical molecular mass of ~18 kDa compared with its experimental monomeric molecular mass of ~16 kDa. **b,** In vitro ε-NAD^+ degradation assays with TIR domain-only protein at different concentrations. WT TIR-APAZ/Ago complex and the catalytic-dead E77A mutant in the presence of target ssDNA were used as the positive and negative controls, respectively. All the assays were performed three times, and the error bars represent the standard deviations. **c,** Arg114 in the center of the TIR tetramer participates in both intrastrand and

interstrand contacts. The four TIR domains are colored as in Fig. 2a. **d,** Superposition of TIR domains in the active and the inactive TIR-APAZ/Ago units. TIR domains are shown in cartoon representation and colored according to B-factors. The extended BB-loops in the active units are more stable than the flipped-in BB-loops in the inactive units and TIR-APAZ/Ago-gRNA ribonucleoprotein (RNP) complex. **e,** Superposition of APAZ domains in the four units of TIR-APAZ/Ago-gRNA-DNA complex (A/A'/B/B') and the RNP complex. **f,** Superposition of TIR-APAZ proteins in the TIR-APAZ/Ago-gRNA-DNA and the RNP complexes. The same color scheme as in Fig. S5e is used.
