# Supplementary material for: Structural insights into mechanisms of Argonaute protein-associated NADase activation in bacterial immunity
Source: Cell Res. 2023 Jun 13;33(9):699–711. doi: 10.1038/s41422-023-00839-7 (PMC10474274; doi:10.1038/s41422-023-00839-7)
Supplement: Supplementary file 6 — Supplementary information, Fig. S6 [file 41422_2023_839_MOESM6_ESM.pdf]

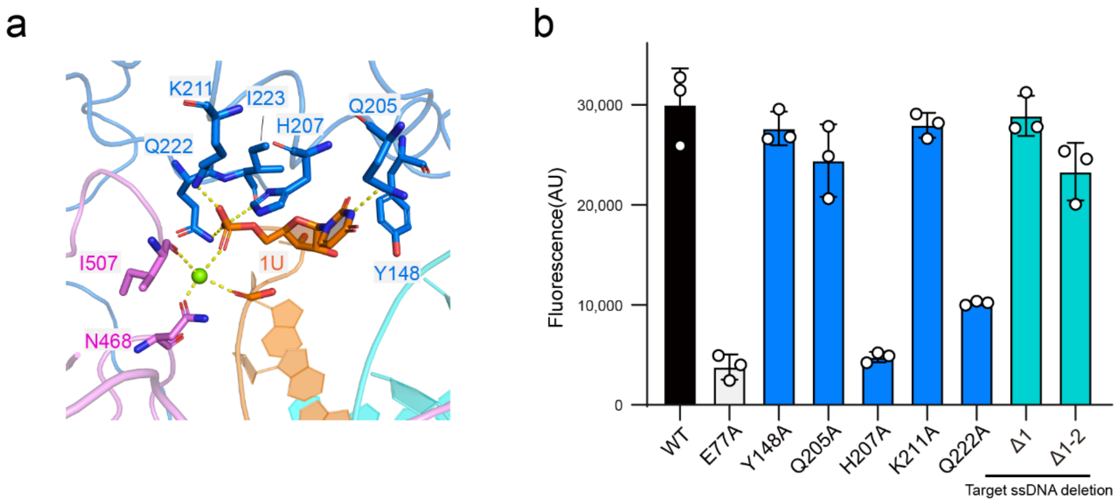

Supplementary information Figure S6. Metal-dependent 5'-phosphate recognition is important for the NADase activity of TIR-APAZ/Ago complex. a, Close-up view of the 5'-phosphate of gRNA and the metal ion binding pocket formed between MID and PIWI domains. The same color scheme as in Fig. 1b is used. **b,** Mutations of the residues in the 5'-phosphate group of gRNA binding pocket affected NAD^+ hydrolysis. Δ1 and Δ1-2 indicate the deletion of the 1'dA and 1'dA-2'dC nt of target ssDNA, respectively. The columns are colored the same as the corresponding residues in Fig. S6a. All assays were performed in triplicate, and the error bars represent the standard deviations.
