# Supplementary material for: Structural insights into mechanisms of Argonaute protein-associated NADase activation in bacterial immunity
Source: Cell Res. 2023 Jun 13;33(9):699–711. doi: 10.1038/s41422-023-00839-7 (PMC10474274; doi:10.1038/s41422-023-00839-7)
Supplement: Supplementary file 7 — Supplementary information, Fig. S7 [file 41422_2023_839_MOESM7_ESM.pdf]

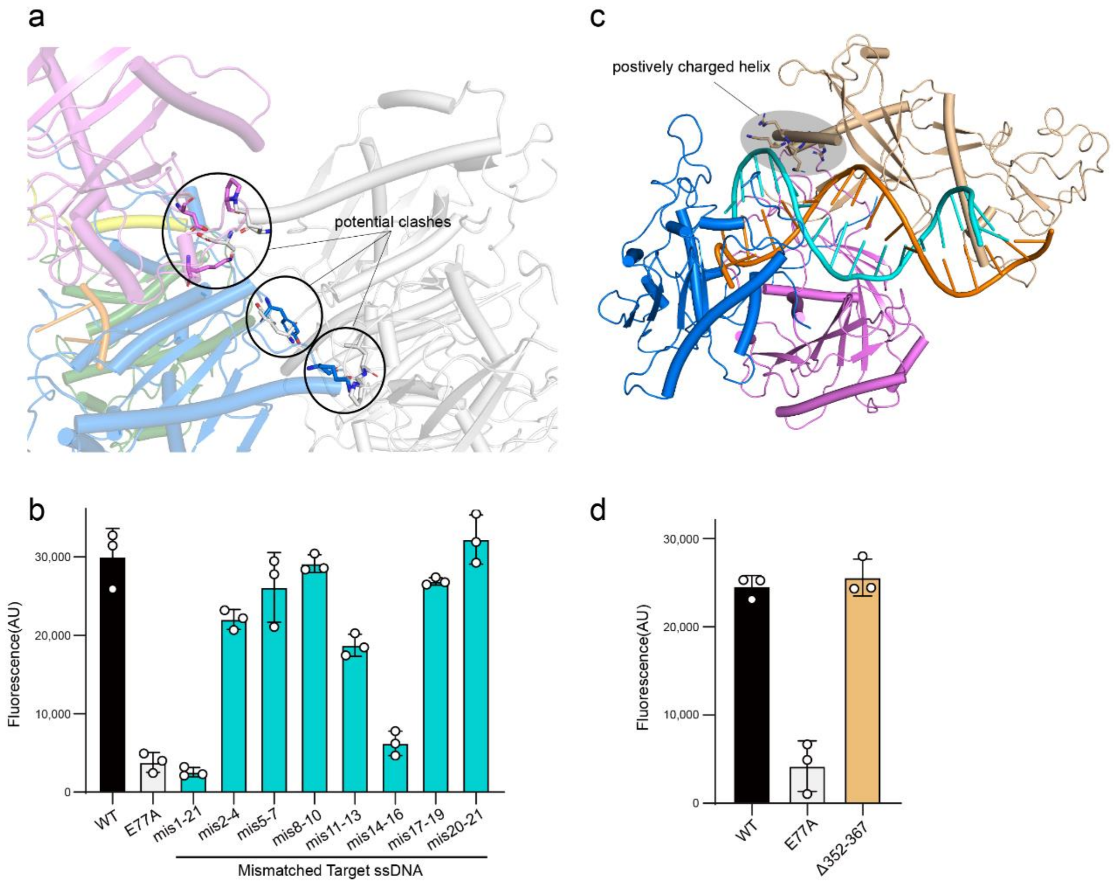

Supplementary information Figure S7. Target ssDNA recognition by the TIR-APAZ/Ago complex. a, Docking of the two Ago proteins in the target-free state (one shown in white, the other colored by domains) into the corresponding positions of those in the target-bound state, and the potential clashes are marked using black circles. **b,** In vitro NAD^+ degradation assays by WT TIR-APAZ/Ago proteins in the presence of mismatched target ssDNA. Mismatches at the central region of target ssDNA, such as 11-16, significantly reduced NAD^+ cleavage. All assays were performed in triplicate, and error bars represent the standard deviations. **c,** A positively charged helix in the APAZ domain is inserted into the gRNA-DNA hybrid. **d,** In vitro NAD^+ degradation assay of the deletion mutation in the positively charged helix of TIR-APAZ/Ago complex. The positively charged helix (aa 352-367) was replaced by a flexible 3xGSA linker, and this mutation had no obvious effect on the NADase activity. All assays were performed in triplicate.
