# Supplementary material for: Structural insights into mechanisms of Argonaute protein-associated NADase activation in bacterial immunity
Source: Cell Res. 2023 Jun 13;33(9):699–711. doi: 10.1038/s41422-023-00839-7 (PMC10474274; doi:10.1038/s41422-023-00839-7)
Supplement: Supplementary file 8 — Supplementary information, Fig. S8 [file 41422_2023_839_MOESM8_ESM.pdf]

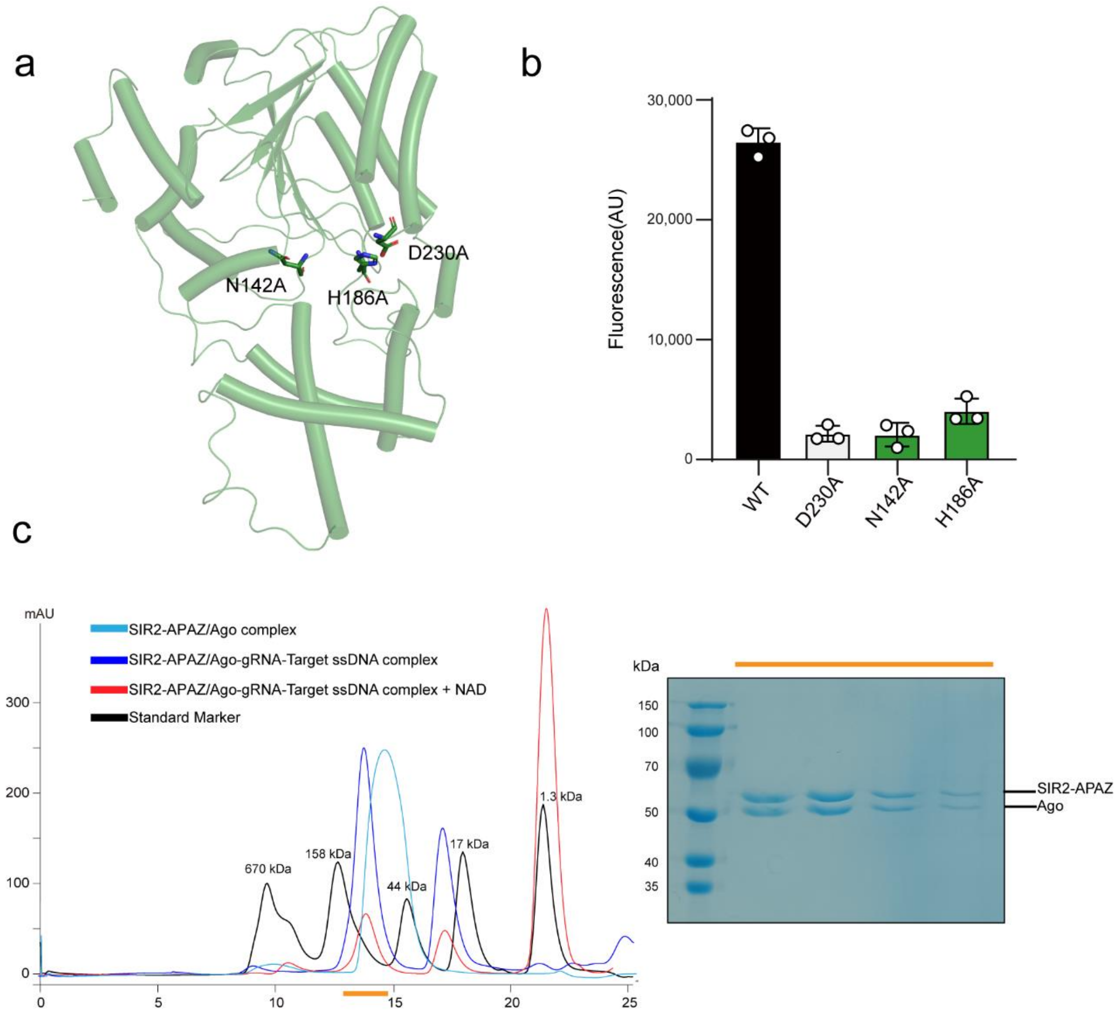

Supplementary information Figure S8. Biochemical characterization of the SIR2-APAZ/Ago system. a, Overall structure of SIR2 domain predicted by AlphaFold. Key residues in the substrate binding pocket are shown as sticks. **b,** In vitro NAD^+ degradation assays by WT and mutant SIR2-APAZ/Ago proteins. Replacement of key residues in the substrate binding pocket significantly impaired the NADase activity of the SIR2-APAZ/Ago system. All assays were performed in triplicate, and error bars represent the standard deviations. **c,** Oligomerization analyses of the SIR2-APAZ/Ago complexes by size exclusion chromatography. Notably, the oligomerization state of SIR2-APAZ/Ago complex was not altered in the presence of NAD^+. The peak of interest was collected and analyzed with SDS-PAGE.
