# Supplementary material for: Structural insights into mechanisms of Argonaute protein-associated NADase activation in bacterial immunity
Source: Cell Res. 2023 Jun 13;33(9):699–711. doi: 10.1038/s41422-023-00839-7 (PMC10474274; doi:10.1038/s41422-023-00839-7)
Supplement: Supplementary file 9 — Supplementary information, Fig. S9 [file 41422_2023_839_MOESM9_ESM.pdf]

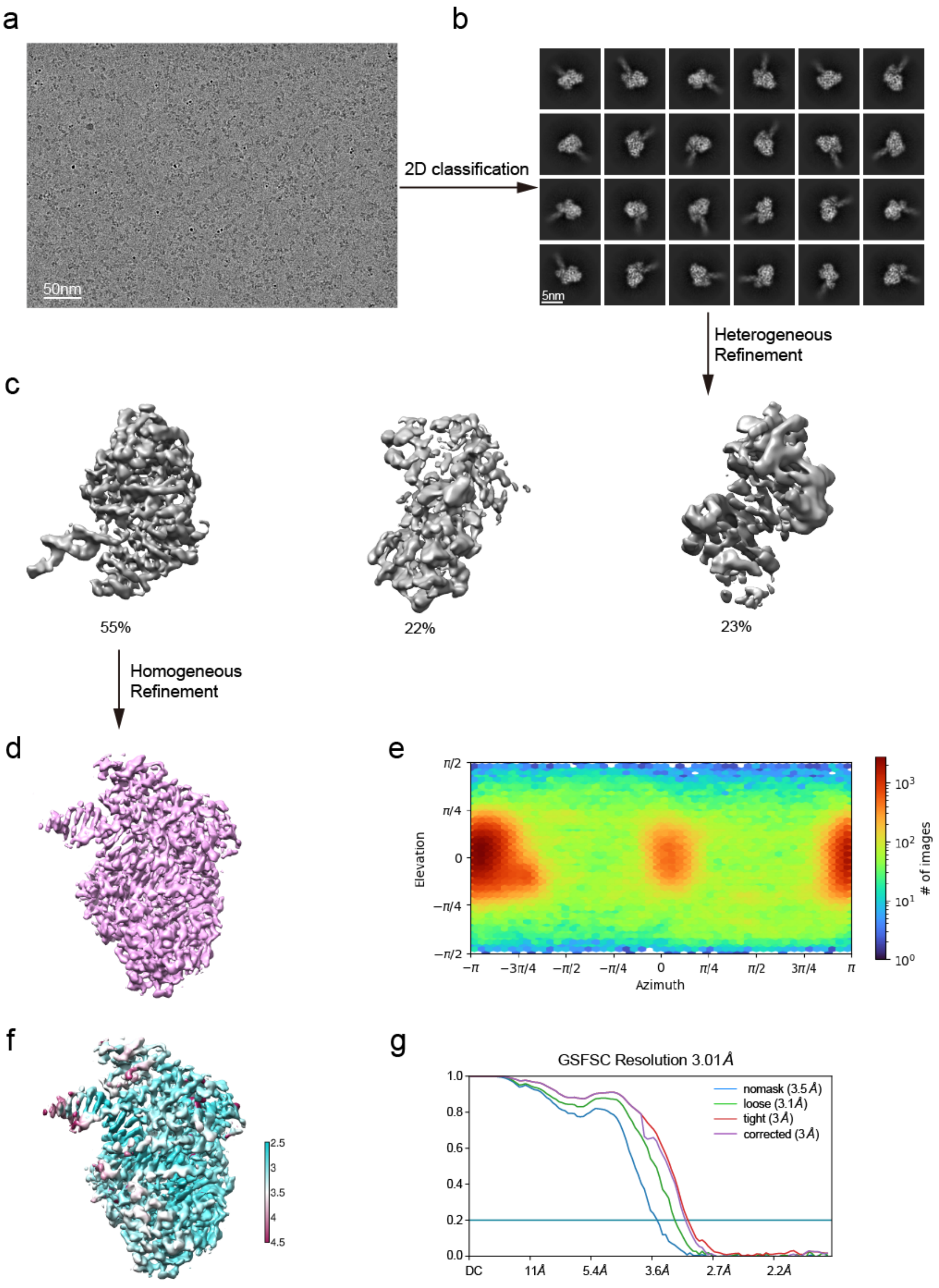

Supplementary information Figure S9. Cryo-EM of reconstruction of the SIR2-APAZ/Ago-gRNA-DNA complex. a-d, The flowchart of the image processing. A representative cryo-EM micrograph (**a**), representative 2D class averages (**b**), 3D classes from Heterogeneous Refinement (**c**) and the final reconstruction (**d**) are shown. **e,** Orientation distribution of particles in the final reconstruction. **f,** Local resolution

analysis. The map is colored according to the local resolution. **g,** FSC plot of the reconstruction. The final resolution is estimated based on the FSC=0.143 cutoff.
