# Supplementary material for: Structural insights into mechanisms of Argonaute protein-associated NADase activation in bacterial immunity
Source: Cell Res. 2023 Jun 13;33(9):699–711. doi: 10.1038/s41422-023-00839-7 (PMC10474274; doi:10.1038/s41422-023-00839-7)
Supplement: Supplementary file 10 — Supplementary information, Fig. S10 [file 41422_2023_839_MOESM10_ESM.pdf]

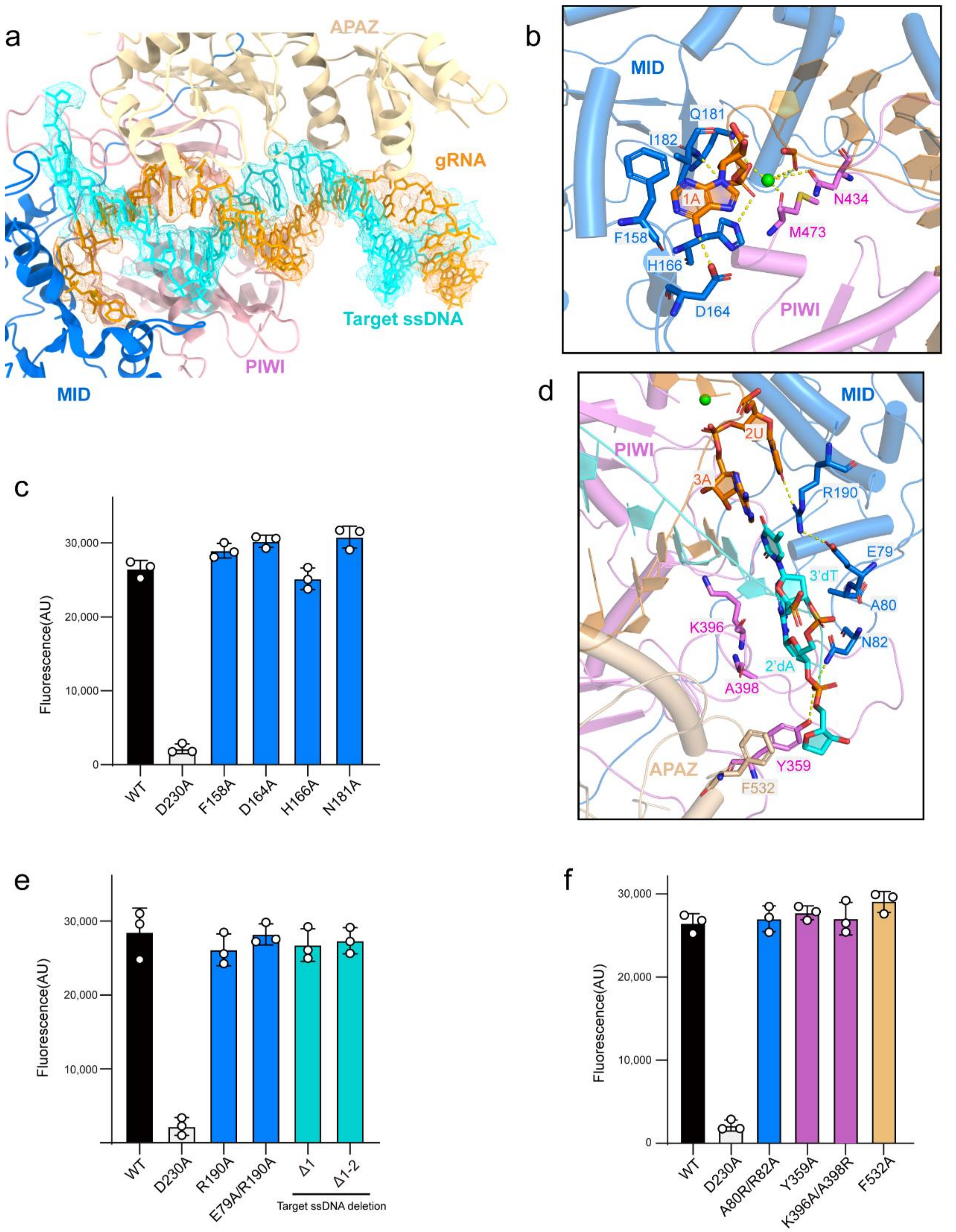

Supplementary information Figure S10. Target ssDNA recognition by the SIR2-APAZ/Ago complex. a, Cryo-EM density and the atomic model of the gRNA-target ssDNA duplex in SIR2-APAZ/Ago-gRNA-DNA quaternary complex. The same color scheme as in Fig. 6b is used. **b,** Close-up view of the binding pocket of the 5'-phosphate group of gRNA. Key residues in the binding pocket are shown as sticks. **c,** NAD^+ hydrolysis by WT and mutant SIR2-APAZ/Ago proteins. Substitutions of key residues

involved in the binding of 5'-phosphate of 1A nucleotide had little effect on the NAD^+ cleavage. The columns are colored the same as the corresponding residues in Fig. S10b. All assays were performed in triplicate, and error bars represent the standard deviations. **d,** Close-up view of the binding pocket of the 2'dA nucleotide on target ssDNA. Key interacting residues in the binding pocket are shown as sticks. **e-f,** Mutations of the residues in the 2'dA-binding pocket did not affect the NAD^+ cleavage. Δ1 and Δ1-2 indicate the deletion of the 1'dT and 1'dT-2'dA nt of target ssDNA, respectively. The columns are colored the same as the corresponding residues in Fig. S10d. All assays were performed in triplicate, and error bars represent the standard deviations.
