# Supplementary material for: Structural insights into mechanisms of Argonaute protein-associated NADase activation in bacterial immunity
Source: Cell Res. 2023 Jun 13;33(9):699–711. doi: 10.1038/s41422-023-00839-7 (PMC10474274; doi:10.1038/s41422-023-00839-7)
Supplement: Supplementary file 11 — Supplementary information, Fig. S11 [file 41422_2023_839_MOESM11_ESM.pdf]

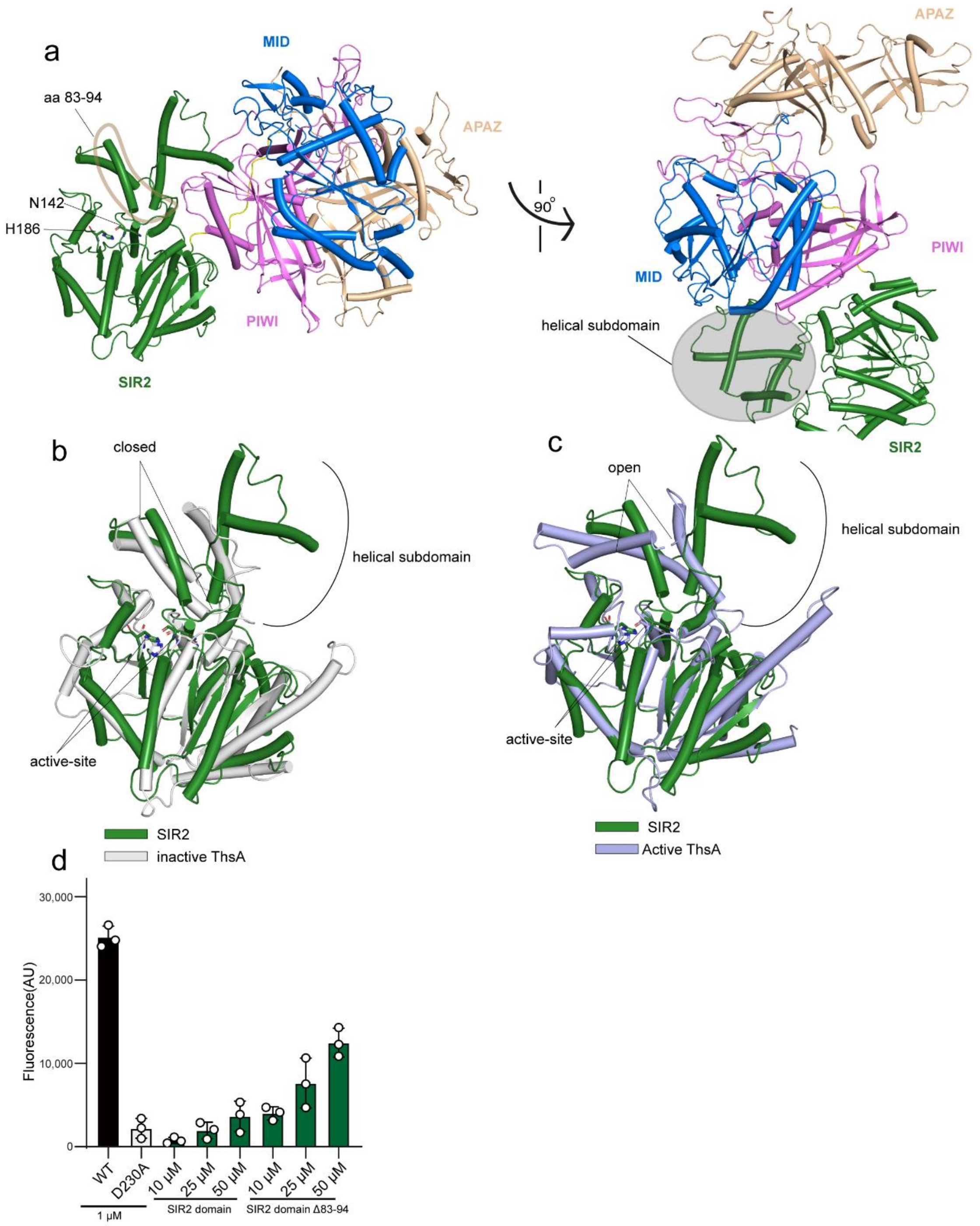

Supplementary information Figure S11. The SIR2 domain is unleashed and activated upon target ssDNA binding. a, The apo SIR2-APAZ/Ago complex structure predicted by AlphaFold. The structure is shown in the same color scheme as in Fig. 6b. **b-c,** Overlaid of the SIR2 domain (green) in the SIR2-APAZ/Ago system and those in the inactive ThsA structure (white, PDB ID: 7UXT) or active ThsA structure (light blue, PDB ID: 6LHX). **d,** In vitro ε-NAD^+ degradation assays of SIR2 domains and truncated SIR2 domains at different concentrations. Δ83-94 indicates

the replacement of residues 83-94 with a linker (GSAGSAG). Mutation of the helical subdomain (Δ83-94) restored the NADase activity of SIR2 domain. All assays were performed in triplicate, and error bars represent the standard deviations.
