# Supplementary material for: Structural insights into mechanisms of Argonaute protein-associated NADase activation in bacterial immunity
Source: Cell Res. 2023 Jun 13;33(9):699–711. doi: 10.1038/s41422-023-00839-7 (PMC10474274; doi:10.1038/s41422-023-00839-7)
Supplement: Supplementary file 12 — Supplementary information, Fig. S12 [file 41422_2023_839_MOESM12_ESM.pdf]

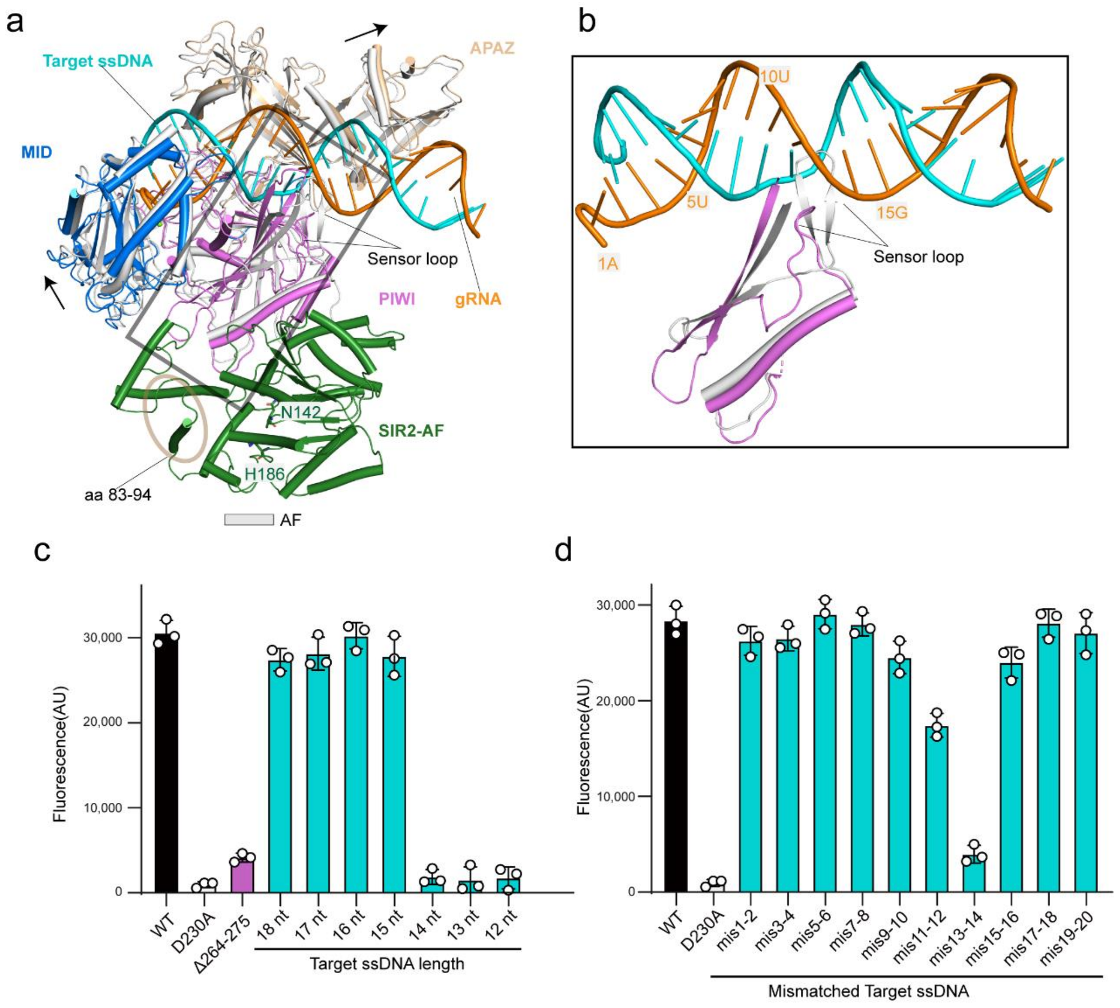

Supplementary information Figure S12. The sensor loop is indispensable for the NADase activity of SIR2-APAZ/Ago system. a, Structural comparison of the SIR2-APAZ/Ago-gRNA-ssDNA quaternary complex with the apo model (green for SIR2 domain and white for the other domains) predicted by AlphaFold. The potential active-site residues N142 and H186 are shown in stick representation. **b,** Close-up view of the conformational change in PIWI domain after target ssDNA binding. The sensor loop in the PIWI domain is indicated by black lines. **c,** In vitro NAD^+ degradation assays by WT or mutant SIR2-APAZ/Ago complexes in the presence of varied target ssDNA. 15 nt ssDNA was required for NADase activity. All assays were performed in triplicate, and the error bars represent the standard deviations. **d,** In vitro NAD^+ degradation assays by WT SIR2-APAZ/Ago proteins in the presence of mismatched target ssDNA. Mismatches at the central region of ssDNA compromised the NADase activity of SIR2-APAZ/Ago system. All assays were performed in

triplicate, and error bars represent the standard deviations.
