# Supplementary material for: Structural insights into mechanisms of Argonaute protein-associated NADase activation in bacterial immunity
Source: Cell Res. 2023 Jun 13;33(9):699–711. doi: 10.1038/s41422-023-00839-7 (PMC10474274; doi:10.1038/s41422-023-00839-7)
Supplement: Supplementary file 13 — Supplementary information, Fig. S13 [file 41422_2023_839_MOESM13_ESM.pdf]

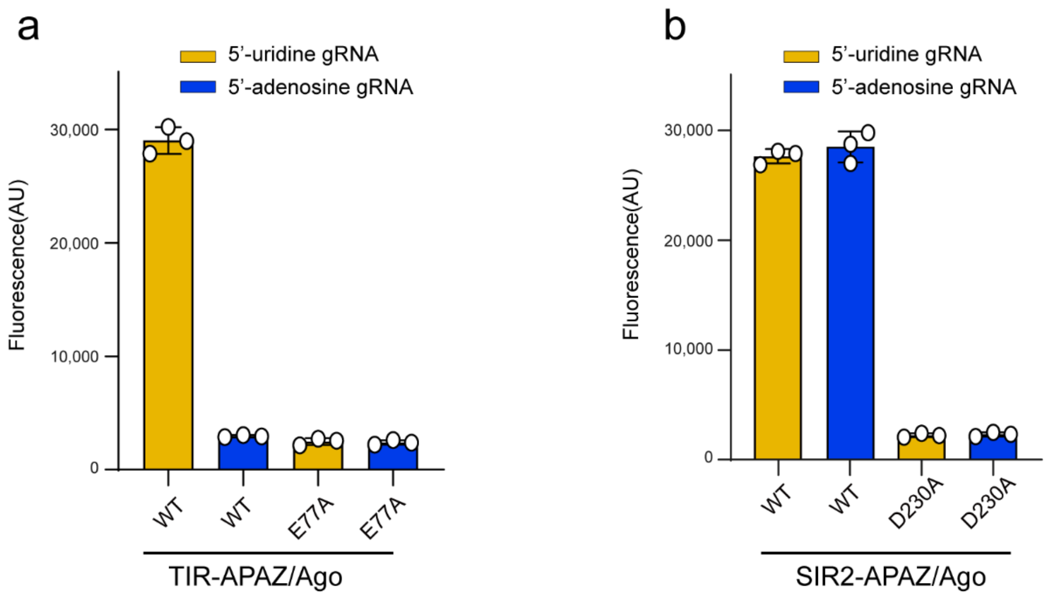

Supplementary information Figure S13. Nucleotide preference for the 5'-end of gRNA. a-b, NADase activities of the TIR-APAZ/Ago system (**a**) and the SIR2-APAZ/Ago system (**b**) when incubated with 5'-uridine or 5'-adenosine gRNA and the corresponding target ssDNA.
