# Supplementary material for: Structural insights into mechanisms of Argonaute protein-associated NADase activation in bacterial immunity
Source: Cell Res. 2023 Jun 13;33(9):699–711. doi: 10.1038/s41422-023-00839-7 (PMC10474274; doi:10.1038/s41422-023-00839-7)
Supplement: Supplementary file 14 — Supplementary information, Table S1 [file 41422_2023_839_MOESM14_ESM.pdf]

Supplementary information, Table S1. Cryo-EM data collection, refinement and validation statistics.

	TIR-APAZ/Ago-gRNA-DNA (EMDB-35240) (PDB 8I87)	TIR-APAZ/Ago-gRNA (EMDB-35241) (PDB 8I88)	SIR2-APAZ/Ago-gRNA-DNA (EMDB-35592) (PDB 8IN8)
**Data collection and processing**			
Magnification	50,000	105,000	105,000
Voltage (kV)	300	300	300
Electron exposure (e–/Å^2)	40	54	54
Defocus range (μm)	-0.5 to -2.5	-1.2 to - 2.2	-1.2 to - 2.2
Pixel size (Å)	0.95	0.85	0.85
Symmetry imposed	*C1*	*C1*	*C1*
Initial particle images (no.)	2,181,364	4,037,142	2,890,897
Final particle images (no.)	369,558	373,126	380,948
Map resolution (Å)	2.95	3.69	3.01
FSC threshold	0.143	0.143	0.143
Map resolution range (Å)	2.5 - 5	3.5 - 5.0	2.5 - 5.0
**Refinement**			
Initial model used (PDB code)	AlphaFold2	AlphaFold2	AlphaFold2
Model resolution (Å)	3.2	3.9	3.2
FSC threshold	0.5	0.5	0.5
Model resolution range (Å)	2.9 - 3.2	3.1 - 3.9	2.9 - 3.2
Map sharpening *B* factor (Å^2)	-99.6	-202.8	-145.8
Model composition			
Non-hydrogen atoms	31066	7287	6663
Protein residues	3519	883	724
Nucleotides	143	2	47
Ligands	4	0	1
B factors (Å^2)			
Protein	71.19	62.68	55.20
Nucleotide	115.40	71.43	76.57
Ligand	20.29		48.76
R.m.s. deviations			
Bond lengths (Å)	0.003	0.003	0.003
Bond angles (°)	0.515	0.655	0.614
Validation			
MolProbity score	2.24	1.78	2.37
Clashscore	8.93	9.42	10.14
Poor rotamers (%)	4.20	0.38	3.63
Ramachandran plot			
Favored (%)	95.78	95.90	93.73
Allowed (%)	4.05	3.99	6.27
Disallowed (%)	0.17	0.11	0
